# Gas6 induces AIM to suppress acute lung injury in mice by inhibiting NLRP3 inflammasome activation and inducing autophagy

**DOI:** 10.3389/fimmu.2025.1523166

**Published:** 2025-02-17

**Authors:** Seonghee Jeong, Kyungwon Yang, Ye‐Ji Lee, Joo-Won Park, Eun-Mi Park, Jihee Lee Kang

**Affiliations:** ^1^ Department of Physiology, College of Medicine, Ewha Womans University, Seoul, Republic of Korea; ^2^ Inflammation-Cancer Microenvironment Research Center, College of Medicine, Ewha Womans University, Seoul, Republic of Korea; ^3^ Department of Biochemistry, College of Medicine, Ewha Womans University, Seoul, Republic of Korea; ^4^ Department of Pharmacology, College of Medicine, Ewha Womans University, Seoul, Republic of Korea

**Keywords:** Gas6, AIM, inflammasome, autophagy, efferocytosis, acute lung injury

## Abstract

**Introduction:**

Growth arrest-specific 6 (Gas6) protein signaling plays a critical role in maintaining immune homeostasis and regulating inflammation. However, novel mechanisms for modulating macrophage activity through the Gas6 axis are being identified. Gas6 enhances the production of apoptosis inhibitor of macrophages (AIM), a protein with potent anti-inflammatory properties. This study investigates whether Gas6-induced AIM suppresses acute lung injury (ALI) in mice by modulating key inflammatory pathways, including inflammasome activation, autophagy, reactive oxygen species (ROS) generation, and efferocytosis.

**Methods:**

ALI was induced in wild-type (WT) and *AIM^−/−^* mice via intratracheal administration of LPS. To evaluate the effects of the Gas6-AIM axis on lung inflammation, recombinant Gas6 (rGas6) was treated intraperitoneally. Inflammatory responses were evaluated using enzyme-linked immunosorbent assay, a cell-sizing analyzer, and Bicinchoninic acid assays. Lung pathology was assessed using hematoxylin-eosin staining. NLRP3 inflammasome activation and autophagy were evaluated using western blot, quantitative real-time PCR, and immunofluorescence. Reactive oxygen species (ROS) levels in alveolar macrophages were measured via fluorescence microscopy, while efferocytosis was assessed in cytospin-stained BAL cells and cultured alveolar macrophages co-cultured with apoptotic Jurkat cells. Additionally, rGas6-mediated effects on NLRP3 inflammasome activation and autophagy were validated in mouse bone marrow-derived macrophages (BMDMs) using siRNAs targeting AIM, Axl, LXRα, or LXRβ.

**Results:**

Proinflammatory cytokine production, neutrophil infiltration, and protein levels in BALF were significantly reduced by rGas6 administration in WT mice but not in *AIM^−/−^* mice. Specifically, rGas6 reduced IL-1β and IL-18 levels, caspase-1 activity, and the production of apoptosis-associated speck-like protein containing a caspase activation and recruitment domain (ASC) in alveolar macrophages. Additionally, rGas6 promoted autophagy and efferocytosis in alveolar macrophages while reducing ROS levels through AIM production. These protective effects were absent in *AIM^−/−^* mice. Furthermore, siRNA-mediated silencing of Axl, LXRα, LXRβ, or AIM reversed the inhibitory effects of rGas6 on NLRP3 inflammasome activation in BMDMs, and AIM was essential for rGas6-induced autophagy.

**Conclusion:**

Gas6-induced AIM production protects against LPS-induced ALI by inhibiting NLRP3 inflammasome activation, enhancing autophagy and efferocytosis, and reducing oxidative stress. These findings highlight the Gas6–AIM axis as a potential therapeutic target for mitigating inflammatory lung diseases.

## Introduction

Acute lung injury (ALI) and acute respiratory distress syndrome (ARDS) are severe clinical conditions characterized by pulmonary edema, excessive inflammatory responses, and increased vascular permeability, resulting in significant morbidity and mortality (1, 2). Despite substantial research efforts, there are no clinically effective pharmacological therapies for ALI/ARDS, underscoring the urgent need for novel therapeutic strategies (1, 3).

The innate immune receptor NOD-like receptor family pyrin domain containing 3 (NLRP3) inflammasome is a critical mediator of inflammatory responses. It is activated by various signals, including damage-associated molecular patterns (DAMPs) and pathogen-associated molecular patterns (PAMPs) (4–6). Upon activation, the NLRP3 inflammasome triggers pyroptosis and caspase-1-mediated proteolytic activation of interleukin-1β (IL-1β) and IL-18, which are key drivers of inflammation (7). Notably, NLRP3 inflammasome activation has been implicated in numerous pulmonary diseases, including respiratory infections, chronic obstructive pulmonary disease, and asthma, as well as in the development of ALI/ARDS (7–12).

Autophagy, a fundamental cellular process responsible for degrading and recycling cellular components, plays a crucial role in modulating inflammatory responses. By mitigating excessive activation of NLRP3 inflammasome, autophagy helps maintain immune homeostasis. This regulation occurs through the removal of inflammasome activators, such as intracellular DAMPs, inflammasome components, and pro-inflammatory cytokines (13). Conversely, impaired autophagy has been linked to hyperinflammation and exacerbated NLRP3 inflammasome activity, emphasizing the intricate relationship between autophagy and inflammasome regulation in the context of inflammatory diseases.

Growth arrest-specific 6 (Gas6) is a multifunctional protein that exerts its biological effects primarily through interactions with TAM receptors, including Tyro3, Axl, and Mer (14). The Gas6/TAM signaling pathway plays diverse roles in inflammation, coagulation, cell survival, proliferation, and the apoptotic cell clearance of (15–20). Notably, the Gas6/TAM system exhibits anti-inflammatory properties, primarily by modulating macrophage activity. Gas6 enhances efferocytosis by activating TAM receptors, particularly Mer and Axl, which are essential for apoptotic cell clearance. This process prevents secondary necrosis and inflammation while promoting macrophage polarization from a pro-inflammatory M1 phenotype to an anti-inflammatory, tissue-reparative M2 phenotype. Furthermore, Gas6-mediated recognition of apoptotic cells enhances the activity of cytokines such as IL-4 and IL-13, thereby directing macrophages toward tissue repair programs. These findings highlight the critical molecular mechanisms by which Gas6 influences macrophage function, including modulation of the IFNAR-STAT1 axis, induction of SOCS3 as a feedback regulator, and integration of apoptotic cell sensing with immune homeostasis (21–24).

We previously demonstrated that Gas6 signaling through the Mer receptor and the LXRα/β pathway promotes the production of apoptosis inhibitor of macrophages (AIM) protein in macrophages (25). AIM, a secreted protein endocytosed by various cell types, facilitates repair processes in several diseases, including ischemic stroke and acute kidney injury (26–31). AIM’s protective effects are mediated through its ability to enhance the phagocytic clearance of DAMPs and debris, thereby mitigating inflammation (29, 31). Recent studies also indicate that AIM promotes M2 macrophage polarization through autophagic mechanisms (32). However, the role of the Gas6–AIM axis in regulating inflammatory responses during ALI remains poorly understood.

This study aimed to determine whether Gas6-induced AIM production suppresses acute lung injury (ALI) in mice by modulating key inflammatory pathways, including inflammasome activation, autophagy, reactive oxygen species (ROS) generation, and efferocytosis. Using wild-type (WT) and *AIM^−/−^* mice, we demonstrate that Gas6-induced AIM inhibits NLRP3 inflammasome activation, enhances autophagy and efferocytosis, and reduces oxidative stress in alveolar macrophages, resulting in decreased lipopolysaccharide (LPS)-induced lung inflammation. *In vitro* experiments confirmed that the Gas6–Axl–LXRα/β signaling pathway is essential for AIM production, which in turn inhibits NLRP3 inflammasome activation in bone marrow-derived macrophages (BMDMs). Among the TAM receptors, Axl was specifically investigated due to its high affinity for Gas6, establishing it as a key mediator in this pathway. Although the precise mechanisms by which Gas6/Axl signaling induces AIM production remain unclear, recombinant AIM (rAIM) reversed the effects of Axl knockdown using siRNAs or the Axl-specific inhibitor BGB324, highlighting the essential role of AIM in this pathway. Notably, AIM was indispensable for rGas6-induced autophagy. These findings underscore the Gas6–AIM axis as a pivotal anti-inflammatory pathway in ALI/ARDS, providing insights into its potential as a therapeutic target for inflammatory lung diseases.

## Materials and methods

### Reagents and antibodies

Mouse rGas6 (986-GS), mouse recombinant AIM (rAIM) (2834-CL-050), LPS (*Escherichia coli* serotype 055:B5, L2880), and adenosine 5’-triphosphate disodium salt (A6419) were acquired from R&D Systems (Minneapolis, MN, USA). BGB324 was purchased from MedchemExpress in Carlsbad, California, USA. The antibodies used for Western blot and immunofluorescence are listed in **Supplementary Table S1**.

### Animal protocols

Six-to-eight-week-old pathogen-free male C57BL/6 mice weighing 19–25 g were acquired from Orient Bio (Sungnam, Korea). *AIM^−/−^* mouse embryos were purchased from the Center for Animal Resources and Development (Kumamoto University) with permission from Prof. Toru Miyazaki at the University of Tokyo. A description of the *AIM^−/−^* mice was provided previously (33). All experiments with WT and *AIM^−/−^* mice were conducted using age-matched males (6–8 weeks). The experimental protocol was endorsed by the Animal Care Committee at Ewha Medical Research Institute (EWHA MEDIACUC past-048-7). Mice were cared for and handled in accordance with the National Institutes of Health Guide for the Care and Use of Laboratory Animals. To induce ALI, LPS (4.5 mg/kg in 30 μl PBS) was administered by pharyngeal aspiration (34). Saline (0.9% NaCl) or rGas6 (50 g/kg in 200 μl PBS) was given intraperitoneally 24 h and 1 h before LPS treatment, and then once daily thereafter (35). Mice were euthanized on day 1, 3, or 6 following LPS treatment.

Three-month-old mail *LXRα^−/−^* mice (C57BL/6), kindly provided by Dr. Soo-Hyun Park (36), were used for the *in vitro* study. Age- and gender-matched WT C57BL/6 mice, purchased from Orient Bio, were used as controls.

### Bronchoalveolar lavage and differential cell counts

After bronchoalveolar lavage (BAL) was conducted following previously established protocols (35), BAL fluid (BALF) samples were centrifuged at 500 × *g* for 5 min at 4°C. Cell pellets were purified and resuspended in phosphate-buffered medium. Cell quantification was performed using an electronic Coulter Counter with a cell-sizing analyzer (Coulter Model ZBI with channelizer 256; Coulter Electronics, Bedfordshire, UK). Neutrophils and alveolar macrophages were identified by their distinct cell diameters. In addition, BAL cytospins were stained with the Diff-Quik kit (Dade Behring, Newark, DE, USA) for differentiation of alveolar macrophages and neutrophils (37, 38). Additionally, we assessed phagocytic indices (38, 39) as: (number of apoptotic bodies per 200 total macrophages) × 100. Following BAL, lung tissues were excised and promptly preserved in liquid nitrogen.

### Preparation and culture of alveolar macrophages

Alveolar macrophages were isolated according to established protocols with minor modifications (37). Initially, suspended alveolar macrophages were confirmed for > 95% viability by trypan blue dye exclusion. Subsequently, cells were seeded in 12-well plates (5 × 10^5^ cells per well) and incubated for 1 h in serum-free X-VIVO 10 medium. After incubation, three consecutive washes were performed to eliminate nonadherent cells. Approximately 90–95% of the cells adhering to the plastic surface exhibited morphological characteristics consistent with macrophages. Immunostaining with Mac3 was conducted to validate the identification and purity of the macrophages. We previously published images of alveolar macrophages obtained with an LSM5 PASCAL confocal microscope (Carl Zeiss, Jena, Germany) (40). For the ELISA assay, alveolar macrophages seeded in 24-well plates (1 × 10^5^ cells per well) and incubated for 1 h in serum-free X-VIVO 10 medium. Following incubation, non-adherent cells were removed, and the adherent cells were cultured for an additional 20 h. Supernatants were then collected and analyzed using the ELISA assay.

### Enzyme-linked immunosorbent assay

ELISA kits were used to determine the concentrations of IL-1β, IL-18, tumor necrosis factor-α (TNF-α), and macrophage inflammatory protein-2 (MIP-2), transforming growth factor-β1 (TGF-β1), and AIM in cell-free BALF and culture supernatants per the manufacturer’s instructions (R&D Systems, Minneapolis, MN, USA).

### Total protein concentration

Protein concentrations in BALF samples served as indicators of blood–pulmonary epithelial cell barrier integrity. Total protein content in BALF was quantified using a BCA protein assay kit following the manufacturer’s protocols (Thermo Scientific, Rockford, IL, USA).

### Western blot analysis

Standard Western blots were conducted using lung tissues and BMDM homogenates. Equivalent amounts of protein were separated by SDS-PAGE (#161-0158, Bio-Rad Laboratories, Hercules, CA, USA) and transferred to nitrocellulose membranes (10600001, GE Healthcare Life Science, Piscataway, NJ, USA) using a wet transfer system (Bio-Rad Laboratories). After blocking with 5% bovine serum albumin (BSA)-TBST or 5% milk-TBST for 1 h, the membranes were incubated overnight with the appropriate primary antibodies, followed by incubation with the corresponding secondary antibody for 1 h at 37°C (**Supplementary Table S1**). The blots were developed using an enhanced chemiluminescence detection kit (Thermo Fisher Scientific, Waltham, MA, USA). Protein bands were visualized using an ImageQuant LAS 4000 mini (GE Healthcare, Chicago, IL, USA), Amersham ImageQuant 800 (Cytiva, Marlborough, MA, USA), or Agfa X-ray films (PDC Healthcare, Valencia, CA, USA). For quantification, the density of specific target bands was normalized against β-actin using ImageJ, version 1.37 (National Institutes of Health, Bethesda, MD, USA).

### Quantitative real-time PCR

Total RNA was extracted from alveolar macrophages, lung tissue, and BMDMs using RNAiso Plus (Takara Bio Inc., Kusatsu, Japan), and cDNA synthesis was conducted using ReverTra Ace™ qPCR RT Master Mix (Toyobo, Osaka, Japan) following the manufacturer’s instructions. SYBR Green-based qRT-PCR was conducted using a QuantStudio™ 3 Real-Time PCR System (Applied Biosystems, Foster City, CA, USA). mRNA levels were normalized to 36B4 mRNA and presented as a fold-change in expression compared with the control group. The primer sequences are listed in **Supplementary Table S2**.

### Confocal microscopic analysis

Alveolar macrophages (1.25 × 10^5^ cells/well) and BMDMs (4 × 10^5^ cells/well) were plated on 24- or 12-well plates with a coverslip. Cells were fixed with 4% paraformaldehyde and permeabilized with 0.5% Triton-X100 in PBS for 10 min. To analyze ASC speck formation, samples were incubated with ASC antibody (1:200) overnight, followed by incubation with goat-antirabbit IgG H&L (Alexa Fluor 488) for 1 h and mounting with 4′,6-diamidino-2-phenylindole (DAPI; Scientific, Waltham, MA, USA). ASC speck formation was analyzed with a Zeiss LSM800 laser-scanning confocal microscope and quantified using Image J. The graph represents the percentage of cells with ASC specks in four distinct areas.

To assess the lysosomal-autophagy axis in alveolar macrophages, culture medium was replaced with RPMI containing 100 nM LysoTracker Red DND-99 (#L7526, ThermoFisher) (41), and cells were incubated at 37°C for 1 h before fixation. The fixed cells were washed with 50 mM NH4Cl in PBS for quenching and then permeabilized with 0.3% Tween-20 in PBS for 10 min. The cells were incubated with 1% bovine serum albumin and 1% normal goat serum in PBS for 3 h and subsequently with anti-LC3B antibody overnight at 4°C, followed by Alexa Fluor 488 goat anti-rabbit IgG as a secondary antibody. Images were captured using a confocal microscope (LSM5 PASCAL, Carl Zeiss, Jena, Germany). Numbers of LC3 and LC3-LysoTracker Red colocalized puncta per cell were determined using a green and red puncta colocalization macro and ImageJ software in threshold images with sizes from 3 to 30 pixels^2^ in more than 30 cells from three independent experiments scored in random fields, as described previously (42). **Supplementary Table S1** contains information on the sources of the antibodies and the dilution ratios.

### Immunohistochemistry

Lung tissues were initially immersed in paraffin, fixed in 10% buffered formalin at room temperature for 48 h, dehydrated, and embedded in paraffin. Sections (4 μm thick) were stained with H&E. Blinded analysis of lung sections was performed using a light microscope. Pathological alterations were scored on a five-point scale according to the degree of neutrophil infiltration into airspace and peribronchiolar space in each image: (0) none (no neutrophil infiltration or a few neutrophils in several alveoli); (1) mild (a few neutrophils scattered in alveolar space in less than 10% of the field); (2) moderate (infiltration of neutrophils in alveolar space in 10–30% of the field); (3) severe (infiltration of neutrophils in alveoli in 30–60% of the field); (4) marked cellular infiltration (neutrophil infiltration in more than 60% of alveoli of the field) (43).

Sections were incubated at room temperature with primary antibodies against F4/80, ATG5, or control rabbit IgG for immunofluorescence analysis. Slides were mounted with VECTASHIELD mounting medium containing DAPI (Vector Laboratories, Burlingame, CA, USA) and imaged using a confocal microscope (LSM5 PASCAL, Carl Zeiss, Jena, Germany). Information regarding antibody sources and dilutions is provided in **Supplementary Table S1**.

### Measurement of reactive oxygen species

To measure intracellular ROS levels, alveolar macrophages were incubated for 30 min at room temperature with PBS containing 5 μM 2′,7′-dichlorofluorescein-diacetate (DCFH_2_-DA). Cells were visualized using a fluorescence microscope (Nikon ECLIPSE TE2000-U, Nikon Instruments Inc., Melville, NY, USA). Fluorescence was monitored every 3 min for 2 h using a multi-detection plate reader (excitation, 485 nm; emission, 520 nm; Synergy™ H1, BioTek). Data were obtained from three wells per experimental group and analyzed using the formula *F_x_ − F_0_*, where *F_x_* is the DCF fluorescence measured at the indicated time, and *F_0_* is the DCF fluorescence measured at the beginning of analysis. Linear regression was performed to determine the rate of ROS generation. For mtROS, cells were stained with 5 μM MitoSOX™ Red Mitochondrial Superoxide Indicator. Each labeled ROS was observed under a fluorescence microscope, and the mean fluorescence intensity was determined using Image J.

### Induction of apoptosis

Human T lymphocyte Jurkat cells were obtained from the American Type Culture Collection (Rockville, MD, USA). Apoptosis was induced by ultraviolet irradiation at 254 nm for 10 min. The cells were then incubated in RPMI-1640 with 10% FBS for 2 h at 37°C and 5% CO_2_ before use. The cells were determined to be 70–80% apoptotic based evaluation of nuclear morphology by light microscopy (38, 44). Apoptosis was confirmed by Annexin V-FITC/propidium iodide (BD Biosciences, San Jose, CA) staining followed by flow cytometric analysis on a FACSCalibur system (BD Biosciences) (44, 45).

### *Ex vivo* phagocytosis assays

Jurkat T cells were fluorescently labeled with PKH67 (green) prior to apoptosis induction according to the manufacturer’s instructions (PKH67-Fluorescent Cell Linker Kits for General Cell Membrane Labelling; Sigma-Aldrich). In brief, Jurkat T cells (10^6^ cells/ml) were washed in serum-free culture medium and resuspended in 2 ml PKH67-containing Diluent C (2 × 10^–6^ M) for 4 min at room temperature. Non-labeled alveolar macrophages (10^5^ cells/ml) from WT and *AIM* ^–/–^ mice treated with saline, LPS, or LPS and rGas6 were plated on coverslips in a 24-well plate. Then, PKH67-labeled apoptotic Jurkat cells were co-cultured with alveolar macrophages at a 10:1 ratio for 90 min at 37°C in 500 μl DMEM. The coverslips were washed twice with PBS to remove non-ingested apoptotic cells. The slides were then fixed with 4% paraformaldehyde and permeabilized with 0.1% Triton X-100 (Sigma-Aldrich). The slides were mounted in VECTASHIELD mounting medium with DAPI (Vector Laboratories, Inc., Youngstown, OH, USA). All slides were imaged using a confocal microscope (LSM5PASCAL). For each condition, more than 200 alveolar macrophages were randomly observed and scored by two independent blinded observers. Each condition was tested in duplicate, and the reader was blinded to the sample identification during analysis. Efferocytosis of alveolar macrophages was determined using the phagocytic index.

### Primary culture of BMDMs and their exposure to stimulants

BMDMs were differentiated from bone marrow myeloid stem cells of C57BL/6, *LXRα* ^−/−^ mice, and WT mice as described previously (46, 47). After 7–10 days in culture with L929 complement DMEM, BMDM differentiation was confirmed by FACS analysis using anti-CD11b. BMDMs (1.5×10^6^ cells/well) were treated with rGas6 (100 ng/ml) or rAIM (1 μg/ml) in 12-well plates for 24 h, followed by LPS (100 ng/ml) for 4 h and ATP, 1 mM or 5 mM) for 1 h. Supernatants were then collected for cytokine analysis and caspas-1 activity measurement.

### siRNA transfection

BMDMs (1.5 × 10^6^ cells/well in six-well plates) were transiently transfected with control siRNA or two types of specific siRNAs targeting AIM, LXRα, LXRβ, or Axl (Bioneer, Daejeon, Korea) at a final concentration of 50 nM using Lipofectamine RNAi MAX (Invitrogen, Carlsbad, CA) per the manufacturer’s instructions. Following overnight transfection, the cells were cultured for 24 h. The siRNA sequences are listed in **Supplementary Table S3**.

### Caspase-1 activity assay

Caspase-1 activity was measured using the bioluminescent Caspase-Glo^®^ 1 Inflammasome Assay (#G9951, Promega, Madison, WI, USA) following the manufacturer’s protocol. For the *in vivo* study, alveolar macrophages (5×10^4^ cells/well) from WT and *AIM^−/−^* mice were seeded in 96-well plates and incubated for 1 h. After incubation, non-adherent cells were then removed, and caspase-1 activity was measured in the adherent cells using the same assay. Additionally, caspase-1 activity was analyzed in cell free-BALF. For the *in vitro* study, supernatants from cultured BMDMs were collected and assessed for caspas-1 activity using the assay.

### Statistics

Data are shown as the mean ± standard error of the mean. Analysis of variance was used for multiple comparisons, and Tukey’s *post hoc* test was used when necessary. A two-tailed Student’s t-test was used for comparisons between two sample means. A *P*-value less than 0.05 was considered statistically significant. All data analyses were conducted using Graph Prism 8 (GraphPad Software Inc., San Diego, CA, US). Every group used for *in vivo* studies contained a minimum of three mice. No animal was excluded prior to randomization and experimental intervention. Sample allocation to different groups was done using a random number method.

## Results

### Gas6-induced AIM production is required for anti-inflammatory response in LPS-induced ALI

We explored the role of Gas6-induced AIM production in the regulation of inflammatory responses during LPS-induced ALI using *AIM^−/−^* and WT mice. Loss of AIM protein in lung tissue from *AIM^−/−^* mice was confirmed by Western blot analysis (**Supplementary Figure S1A**). Similar to our previous *in vitro* findings (25), we observed increased *AIM* mRNA expression in alveolar macrophages and lung tissue starting at day 1 and persisting until day 6 following either administration of either recombinant Gas6 (rGas6) or LPS (**Supplementary Figure S1B, C**). Notably, mice treated with LPS and rGas6 showed enhanced AIM mRNA levels at day 3 post-LPS treatment compared with mice treated with either rGas6 or LPS alone. Additionally, AIM protein levels in culture supernatants of alveolar macrophages and BALF were markedly increased at day 3 post-LPS treatment in WT mice treated with LPS and rGas6 compared with those in mice treated with rGas6 or LPS alone (**Supplementary Figures S1D, E**). By contrast, AIM protein expression was undetectable in all samples from *AIM^−/−^* mice treated with saline (control), rGas6, LPS, or LPS and rGas6.

**Figure 1:**
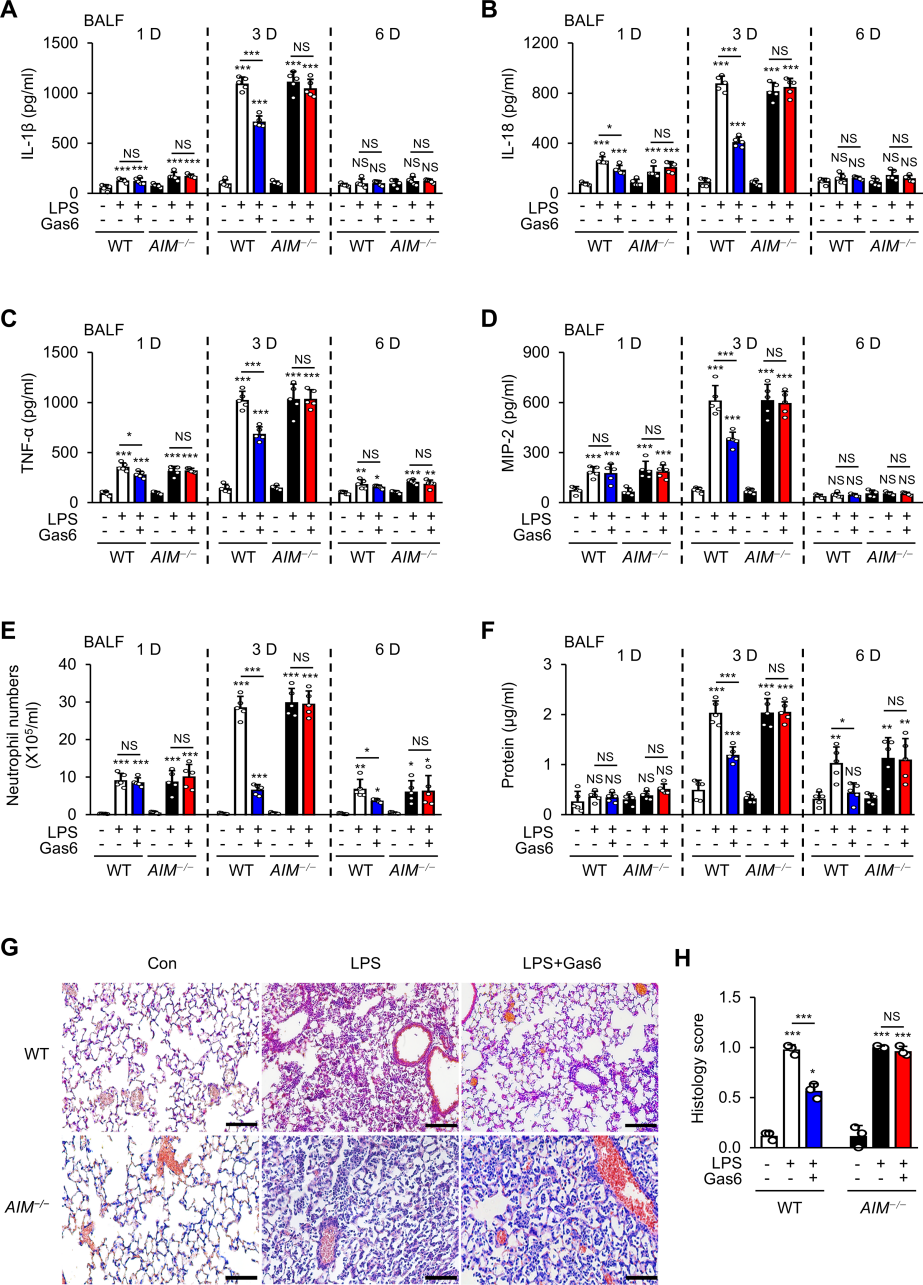
Anti-inflammatory effects of rGas6 in LPS-induced acute lung injury are reversed in *AIM* ^−/−^ mice. **(A–D)** ELISA was performed to quantify the abundance of IL-1β, IL-18, TNF-α, and MIP-2 in bronchoalveolar lavage fluid (BALF). **(E)** Numbers of neutrophils and **(F)** levels of proteins in BALF were determined. **(G)** Hematoxylin-eosin stains of lung sections at day 3 after LPS treatment (original magnification: ×200). Scale bars: 20 μm. Representative images were obtained from three mice per group. **(H)** Histology scores of lung inflammation at day 3 after LPS treatment. Where indicated, WT and *AIM* ^−/−^ mice were intraperitoneally administered rGas6 (50 μg/kg) 1 day before intratracheal instillation with LPS (4.5 mg/kg) and then once daily thereafter. Animals were euthanized at 1, 3, or 6 days after LPS challenge. Values represent the mean ± standard error of three **(G, H)** or five **(A–F)** mice per group. NS, not significant; *P < 0.05, **P < 0.01, and ***P < 0.001 compared with saline control or as indicated.

Next, we examined whether rGas6 attenuates LPS-induced pulmonary inflammatory responses by modulating AIM production. Pulmonary inflammatory responses, including proinflammatory cytokine production, peaked at day 3 post-LPS treatment (**Figures 1A–D**). Administration of rGas6 reduced levels of proinflammatory cytokines such as IL-1β, IL-18, TNF-α, MIP-2 in BALF from WT mice at day 3 post-LPS treatment. We analyzed neutrophil numbers and total protein levels in BALF as markers of inflammatory ALI with enhanced alveolar permeability. In WT mice, neutrophil numbers and total protein content peaked at day 3 post-LPS treatment. Administration of rGas6 reduced these inflammatory responses at days 3 and 6 post-LPS treatment (**Figures 1E, F**); however, the inhibitory effects of rGas6 on inflammatory cytokine production, neutrophil recruitment, and BALF protein content were not shown in *AIM^−/−^* mice. Our previous experiments demonstrated that basal levels of these inflammatory cytokines, neutrophil counts, and protein levels in BALF from naïve lungs after rGas6 administration were similar to those after saline administration (35). Because the maximal level of neutrophils in BALF was observed on day 3 post-LPS treatment, we conducted hematoxylin and eosin (H&E) staining on sections of lung tissue from mice at this time point. Histological findings revealed that rGas6 reduced LPS-induced accumulation of inflammatory cells in alveolar spaces and lung parenchyma. Furthermore, WT mice treated with LPS and rGas6 showed only mild destruction of alveolar structure and reduced histological scores of lung inflammation compared with mice treated with LPS alone (**Figures 1G, H**); however, these beneficial effects of rGas6 treatment were reversed in *AIM^−/−^* mice.

### AIM is required for rGas6 to inhibit NLRP3 inflammasome activation

**Figure 2:**
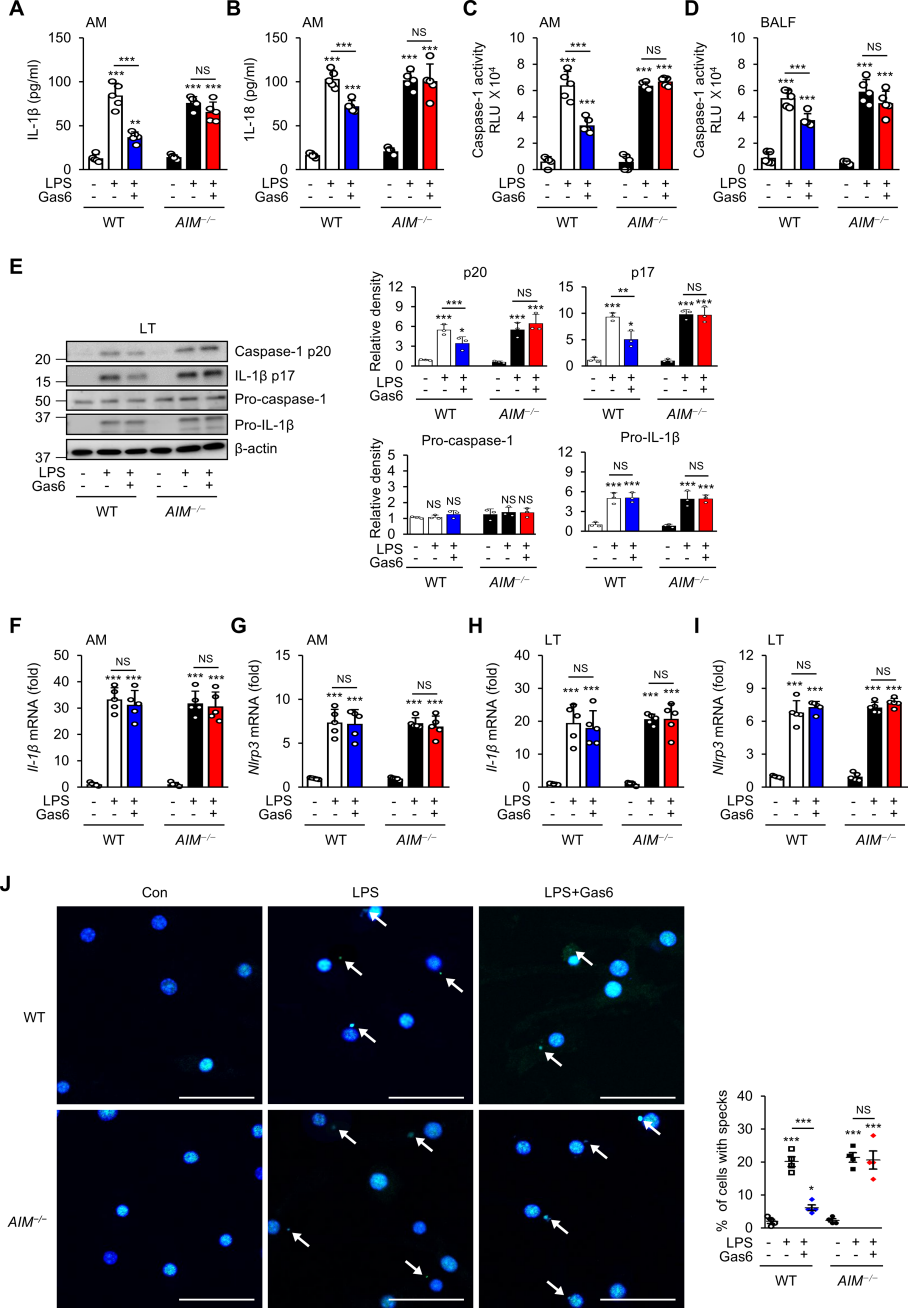
Inhibitory effects of rGas6 on NLRP3 activation in LPS-induced ALI are reversed in *AIM* ^−/−^ mice. **(A, B)** ELISA was performed to quantify the abundance of IL-1β and IL-18 in culture supernatants of alveolar macrophages. **(C, D)** Caspase-1 activity was measured in cultured alveolar macrophages and bronchoalveolar lavage fluid (BALF). **(E)** Left: Immunoblot analysis of the indicated proteins in lung tissue. Right: The relative densitometric intensity was determined for each band and normalized to β-actin. **(F–I)** qRT-PCR analysis of *Il-1β* and *Nlrp3* mRNA levels in alveolar macrophages and lung tissue. **(J)** Left: Representative immunofluorescence confocal microscopic images of ASC specks in alveolar macrophages. DNA is stained blue and ASC green. Arrows point to ASC specks. Original magnification: 200 ×. Scale bars: 50 μm. Right: Quantification of the percentage of cells with ASC specks (4 × 200 cells/nuclei [DAPI-stained], analyzed with ImageJ). Where indicated, WT and *AIM^−/−^* mice were intraperitoneally administered rGas6 (50 μg/kg) 1 day before intratracheal instillation with LPS (4.5 mg/kg) and then once daily thereafter. Animals were euthanized on day 3 after LPS challenge. Values represent the mean ± standard error of three **(E)**, four **(J)**, or five **(A–D, F–I)** mice per group. NS, not significant; *P < 0.05, **P < 0.01, and ***P < 0.001 compared with saline control or as indicated.

Recent evidence indicates that the NLRP3 inflammasome and its regulated cytokines are critical for ALI development (9–11). To investigate whether the Gas6–AIM axis inhibits inflammasome activation following LPS treatment, we analyzed the levels of inflammasome-related cytokines IL-1β and IL-18 in the culture supernatants of alveolar macrophages, as well as caspase-1 activity in both cultured alveolar macrophages and BALF, from WT and *AIM^−/−^* mice at day 3 post-LPS treatment. Administration of rGas6 reduced IL-1β and IL-8 levels in supernatants of alveolar macrophages (**Figures 2A, B**) and markedly reduced caspase-1 activity in supernatants of alveolar macrophages and BALF from WT mice at day 3 post-LPS treatment (**Figures 2C, D**), whereas these reductions were not evident in *AIM^−/−^* mice. Administration of rGas6 suppressed the levels of mature caspase-1 (p20) and IL-1β (p17) in lung tissue lysates on day 3 after LPS treatment in WT mice, but this effect was not observed in *AIM^−/−^* mice (**Figure 2E**). However, rGas6 did not influence LPS-induced increases in pro-IL-1β expression in lysates from both WT and *AIM^−/−^* mice. The levels of pro-caspase-1 in lung tissue lysates from both WT and *AIM^−/−^* mice remained similar to basal levels observed in control saline-treated mice, irrespective of LPS treatment, with or without rGas6 administration. Furthermore, rGas6 did not affect *IL-1β* and *Nlrp3* mRNA expression in alveolar macrophages and lung tissue lysates from WT and *AIM^−/−^* mice following LPS treatment (**Figures 2F–I**). Immunocytochemical analysis showed that rGas6 inhibited ASC speck formation, indicating inflammasome activation, in alveolar macrophages from WT mice at day 3 post-LPS treatment (**Figures 2J**); however, this effect was not shown in *AIM^−/−^* mice.

### The inhibitory effect of Gas6 on NLRP3 inflammasome activation in BMDMs is mediated by AIM

We conducted a series of *in vitro* experiments to confirm that AIM mediates Gas6-induced inhibition of NLRP3 inflammasome activation. Consistent with data from a previous study (48), treatment with 100 ng/ml rGas6 reduced IL-1β and IL-18 production as well as caspase-1 activity in BMDMs stimulated with 100 ng/ml LPS and 1 mM ATP (**Supplementary Figures S2A–C**). Additionally, rGas6 reduced the levels of mature caspase-1 p20 and IL-1β p17 released by BMDMs (**Supplementary Figure S2D**) but did not affect the protein levels of pro-caspase-1 and pro-IL-1β in BMDM lysates. LPS/ATP-induced increases in *IL-1β*, *IL-18*, and *NLRP3* mRNA expression were not inhibited by rGas6 (**Supplementary Figures S2E–G**). Immunocytochemical staining confirmed that rGas6 inhibited inflammasome activation-mediated ASC speck formation (**Supplementary Figure S2H**).

**Figure 3:**
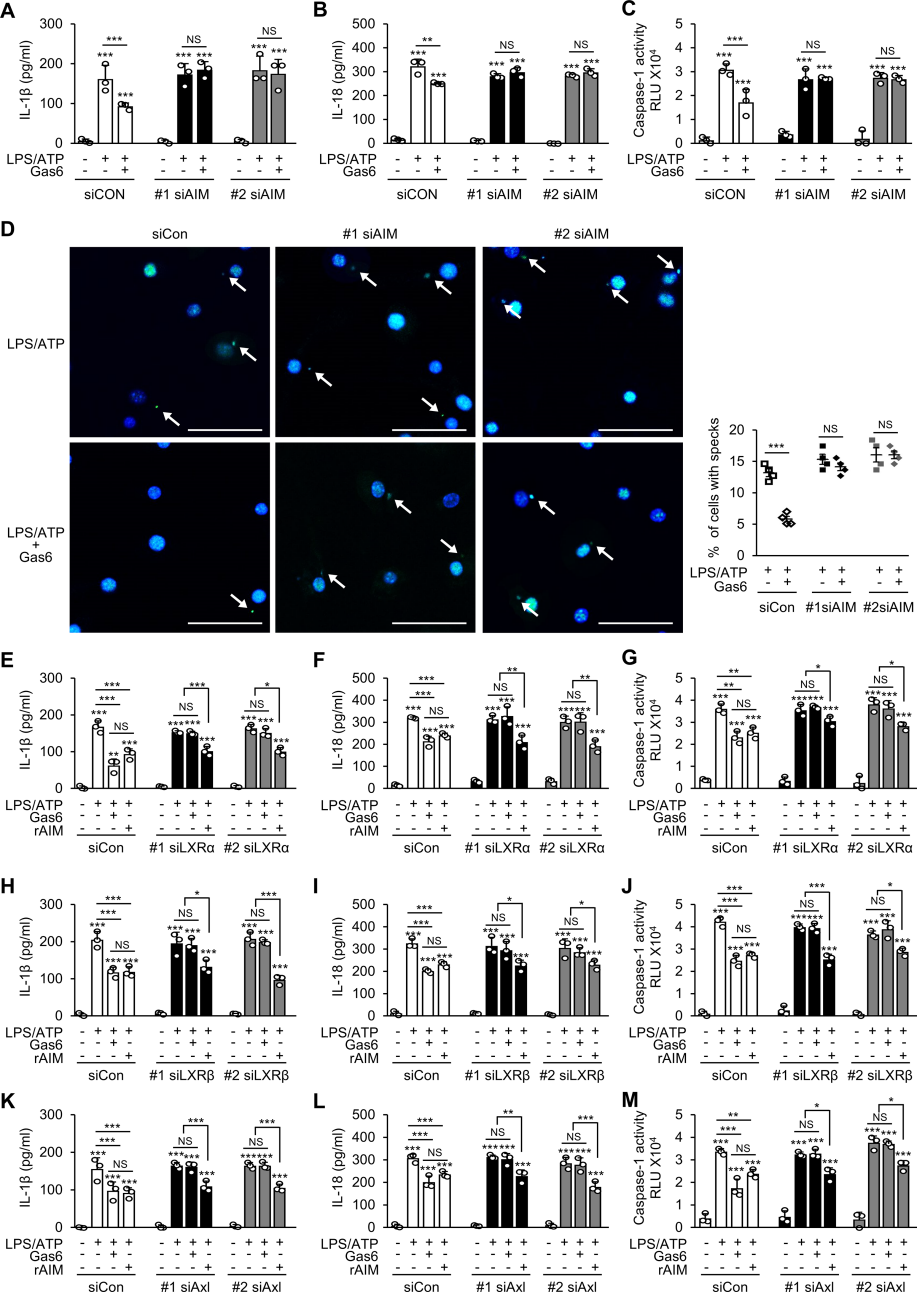
The Gas6–Axl–LXRα/β signaling pathway inhibits NLRP3 inflammasome activation in BMDMs by promoting AIM production. ELISA was performed to quantify the abundance of IL-1β **(A, E, H, K)** and IL-18 **(B, F, I, L)** in culture supernatants of bone marrow-derived macrophages (BMDMs). **(C, G, J, M)** Caspase-1 activity was measured in culture supernatants of BMDMs. **(D)** Left: Representative immunofluorescence confocal microscopic images of ASC specks (green). Arrows point to ASC specks. Original magnification: 200 ×. Scale bars: 50 μm. Right: Quantification of the percentage of cells with ASC specks (4 × 200 cells/nuclei [DAPI-stained], analyzed with ImageJ). BMDMs were transfected with two types of siRNAs for AIM **(A–D)**, LXRα **(E–G)**, LXRβ **(H–J)**, or Axl **(K–M)** and then treated with rGas6 (100 ng/ml) or rAIM (1 μg/ml) for 24 h, followed by LPS (100 ng/ml) for 4 h and ATP [1 mM in **(A–C)** and **(E–M)** or 5 mM in **(D)**] for 1 h. Values represent the mean ± standard error of three **(A–C)**, **(E–M)** or four **(D)** independent experiments. NS, not significant; *P < 0.05, **P < 0.01, and ***P < 0.001 compared with control or as indicated.

To validate the role of AIM in mediating the inhibitory effects of Gas6 on inflammasome activation, we knocked down AIM expression in BMDMs using two types of siRNAs (**Supplementary Figure S3A**). AIM knockdown reversed the inhibitory effects of Gas6 on IL-1β and IL-18 production and caspase-1 activity (**Figures 3A–C**) and ASC speck formation (**Figure 3D**) in LPS/ATP-stimulated BMDMs. Consistent with our *in vivo* results, these results indicate that AIM production was required for Gas6 signaling-induced inhibition of NLRP3 inflammasome activation in BMDMs.

### AIM is essential for Gas6–Axl–LXRα/β signaling to inhibit NLRP3 inflammasome activation in BMDMs

Gas6 signaling induces AIM production by upregulating LXRα/β signaling in BMDMs (25). We examined whether knockdown of LXRα or LXRβ (**Supplementary Figures S3B, C**) reverses the inhibitory effects of Gas6 on IL-1β and IL-18 production and caspase-1 activation in BMDMs. As expected, the inhibitory effects of Gas6 on LPS/ATP-induced IL-1β and IL-18 production and caspase-1 activity were not shown in BMDMs transfected with two types of siRNAs specific for LXRα (**Figures 3E–G**) or LXRβ (**Figures 3H–J**). Among the TAM receptors, Gas6 has the highest affinity for Axl. Therefore, we examined whether inhibition of Gas6–Axl signaling reverses the inhibitory effects of Gas6 on inflammasome activation in BMDMs. Knockdown of Axl in BMDMs (**Supplementary Figure S3D**) with two types of siRNAs abolished the inhibitory effects of Gas6 on LPS/ATP-induced IL-1β and IL-18 production and caspase-1 activity (**Figures 3K–M**). Notably, treatment with 1 μg/ml rAIM resulted in consistent suppression of LPS/ATP-induced IL-1β and IL-18 production and caspase-1 activation in BMDMs, regardless of the knockdown of LXRα, LXRβ, or Axl (**Figures 3E–M**). Similarly, the Gas6-induced suppression was abolished when BMDMs were pretreated with the Axl specific inhibitor BGB 324 (1 μg/ml) or in LXRα-deficient BMDMs (**Supplementary Figures S4A–G**). However, treatment with 1 μg/ml rAIM consistently inhibited IL-1β and IL-18 production as well as caspase-1 activation, irrespective of the presence of Axl specific inhibitor or the absence of LXRα. Collectively, these findings suggest that AIM production through the Gas6–Axl–LXRα/β pathway plays a crucial role in inhibiting NLRP3 inflammasome activation in BMDMs.

### AIM mediates autophagy induction by rGas6

**Figure 4:**
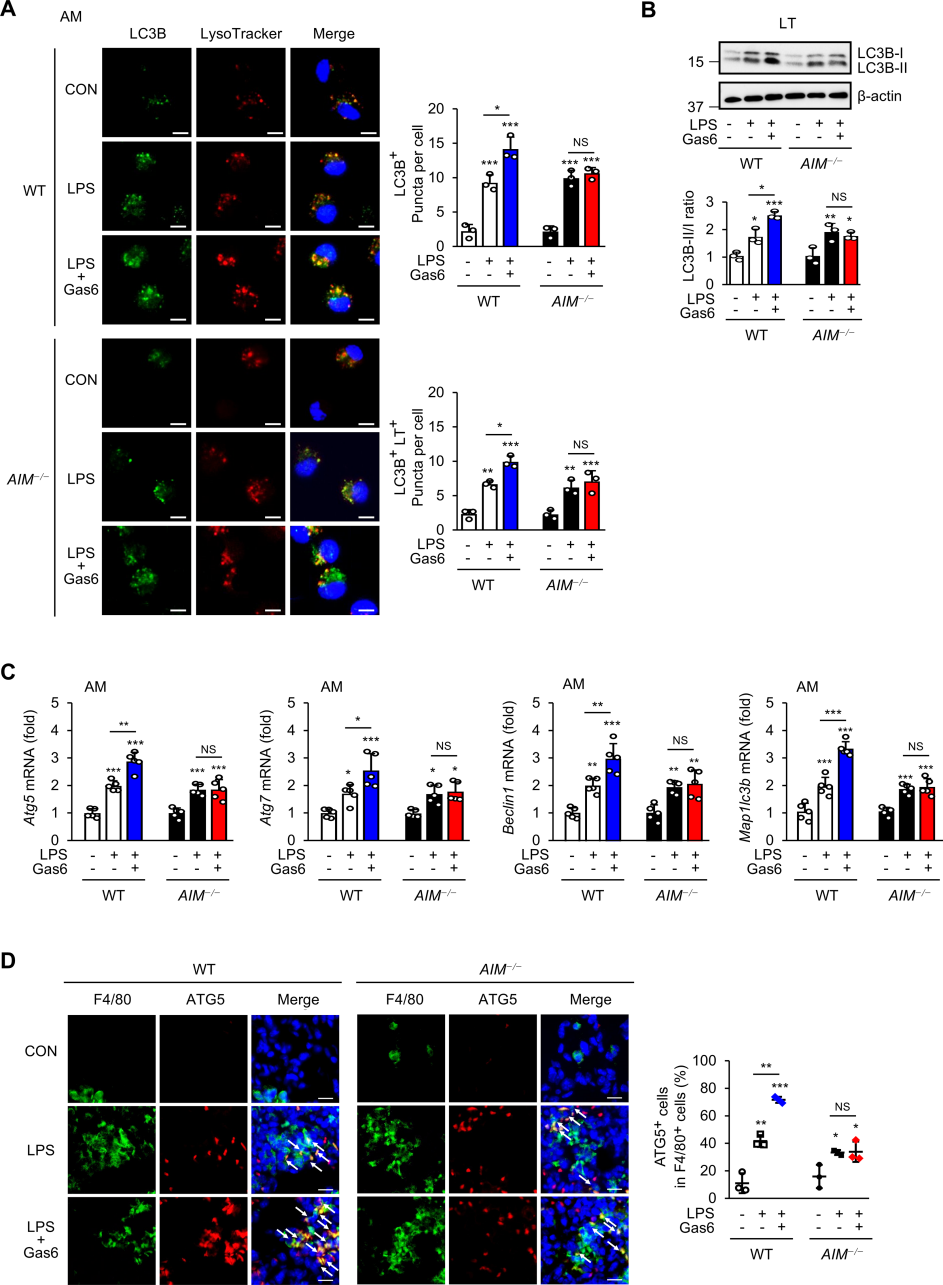
rGas6 promotes autophagy induction mediated by AIM in LPS-induced ALI. **(A)** Left: representative confocal microscopy images of alveolar macrophages showing LC3 (green) and LysoTracker Red staining and colocalization in yellow (merged). Original magnification: 400 ×. Scale bars: 10 μm. Right graphs: Mean ± standard error of the numbers of LC3 puncta per cell (LC3B^+^ puncta/cell) and LC3-LysoTracker Red colocalized puncta per cell (LC3B^+^ LT^+^ puncta/cell), including at least 50 cells scored in random fields. **(B)** Immunoblot analysis of LC3B-I and LC3B-II levels in lung tissue lysates (*upper*). The ratios of LC3B-II to LC3B-I are shown (*lower*). **(C)** qRT-PCR analysis of the autophagy-related genes *Atg5*, *Atg7*, *Becn1*, and *Map1lc3b* in alveolar macrophages. **(D)** Immunohistochemistry of lung sections from WT and *AIM^−/−^* mice stained with anti-F4/80 antibody and anti-ATG5 antibody (ATG5). Right: Quantification of ATG^+^ cells among F4/80^+^ cells. The data are represented as the mean ± standard error from three mice per group. DAPI staining was performed to visualize nuclei. Original magnification: 400 ×. Scale bars: 50 μm. Where indicated, WT and *AIM* ^−/−^ mice were intraperitoneally administered rGas6 (50 μg/kg) 1 day before intratracheal instillation with LPS (4.5 mg/kg) and then once daily thereafter. Animals were euthanized on day 3 after LPS challenge. Values represent the mean ± standard error of three **(A, B, D)** or five **(C)** mice per group. NS, not significant; *P < 0.05, **P < 0.01, and ***P < 0.001 compared with saline control or as indicated.

Gas6 and AIM both signal to induce autophagy (32, 48). We examined whether AIM is required for rGas6 to promote autophagy in alveolar macrophages and attenuate LPS-induced ALI. We quantified microtubule-associated protein 1 light chain 3B (LC3B) puncta to assess autophagy vesicle formation and used the acidotropic fluorescent dye LysoTracker Red to assess LC3B fusion with lysosomes in alveolar macrophages from mice at day 3 post-LPS treatment with or without rGas6 administration. In WT mice, rGas6 enhanced LC3B puncta formation and colocalization with LysoTracker Red compared with LPS treatment alone (**Figure 4A**). These effects of rGas6 were not observed in *AIM^−/−^* mice. In addition, we examined the LC3B-II status by Western blot as an autophagy marker in total lung tissue lysates (**Figure 4B**). These assays revealed that rGas6 enhanced LC3B-II expression and increased the LC3B-II/-I ratio compared with LPS treatment alone in WT mice but not in *AIM^−/−^* mice.

Autophagy-related 5 (ATG5), ATG7, Beclin 1, and LC3 proteins are essential for autophagy induction (49). We monitored their mRNA transcripts by qRT-PCR in alveolar macrophages from WT and *AIM^−/−^* mice post-LPS treatment with or without rGas6 administration. Administration of rGas6 increased the mRNA levels of *Atg5*, *Atg7*, *Becn1*, and *Maplc3b* in alveolar macrophages from WT mice at day 3 post-LPS treatment (**Figure 4C**); however, these increases were not shown in *AIM^−/−^* mice. These data suggest that *AIM^−/−^* mice are not able to inhibit LPS-induced pulmonary inflammation and NLRP3 inflammasome activation because of deficits in Gas6-induced AIM production, which normally mediates autophagy induction. Supporting this idea, immunohistochemical staining with anti-ATG5 antibody showed that WT mice contained higher numbers of ATG5-expressing F4/80^+^ macrophages in lung tissues compared with *AIM^−/−^* mice at day 3 after treatment with LPS and rGAS6 (**Figure 4D**).

**Figure 5:**
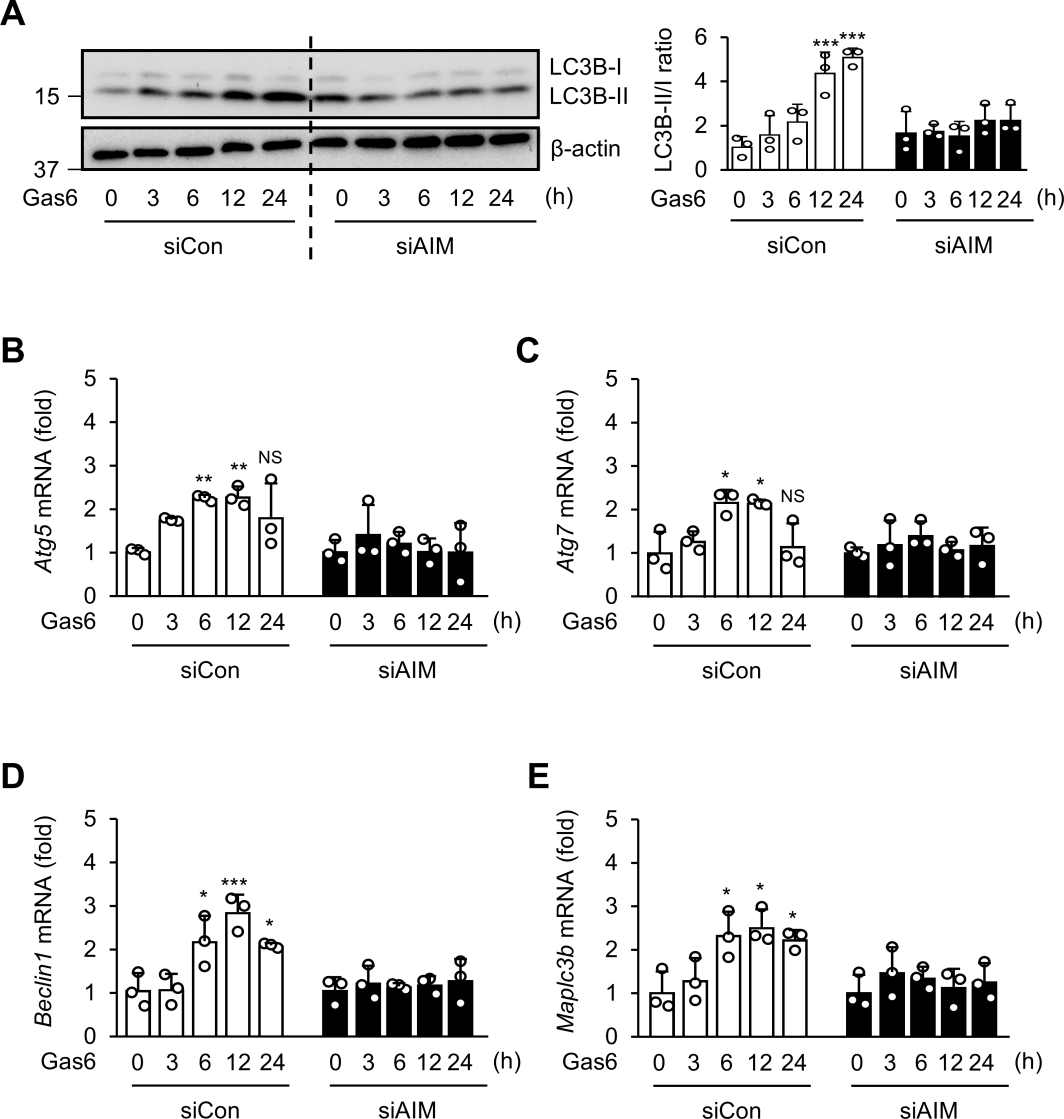
AIM is required for autophagy induction by rGas6 in BMDMs. **(A)** Left: Immunoblot analysis of LC3B-I and LC3B-II levels in BMDMs. Right: The ratios of LC3B-II to LC3B-I are shown. **(B–E)** qRT-PCR analysis of autophagy-related genes *Atg5*, *Atg7*, *Becn1*, and *Map1lc3b* in BMDMs. BMDMs were transfected with control or AIM siRNA and then treated with rGas6 (100 ng/ml) for 3–24h. Values represent the mean ± standard error of three independent experiments. NS, not significant; *P < 0.05, **P < 0.01, and ***P < 0.001 compared with control or as indicated.

To confirm that AIM is required for Gas6 to induce autophagy, we transfected BMDMs with AIM-specific siRNA for 24 h. Treatment with rGas6 resulted in a time-dependent increase in conversion of LC3B-I to LC3BII in control BMDMs, indicating increases in autophagy induction (**Figure 5A**); however, no change in the LC3B-II/-I ratio was observed in BMDMs with AIM knockdown. Additionally, we assessed mRNA expression of *Atg5*, *Atg7*, *Becn1*, and *Map1lc3b* in BMDMs following rGas6 treatment. The induction of these genes was enhanced in control BMDMs, but not in AIM-knockdown BMDMs, following rGas6 treatment (**Figures 5B–E**).

### AIM contributes to reducing ROS production and inducing efferocytosis by rGas6 administration in ALI

**Figure 6:**
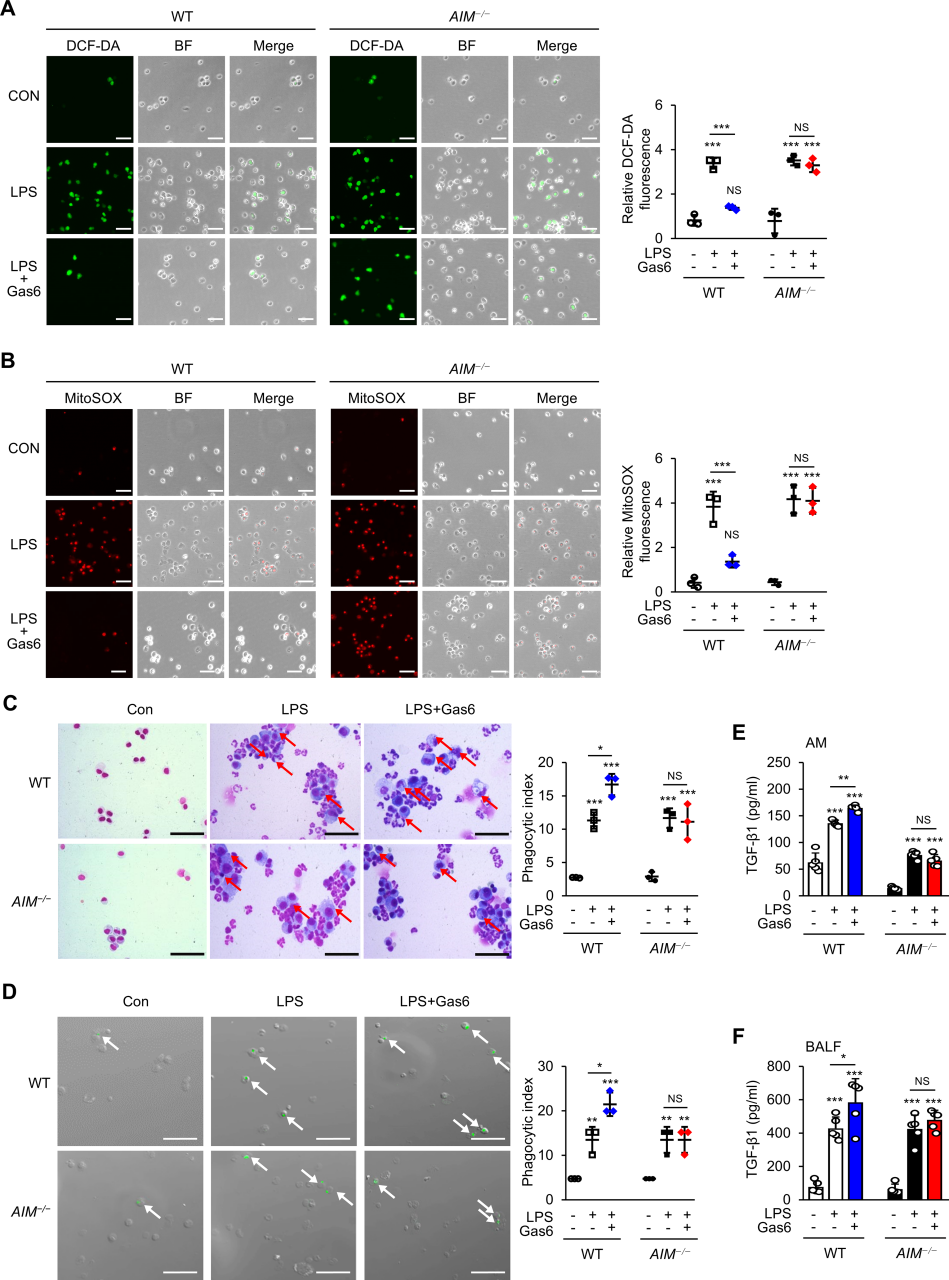
rGas6 requires AIM to inhibit ROS production and enhance efferocytosis and TGF-β production by alveolar macrophages in ALI. **(A, B)** Left: Representative immunofluorescence images of cells. Original magnification: 200 ×. Scale bars: 50 μm. Representative results from three independent experiments are shown. Isolated alveolar macrophages were stained with 5 μM H_2_DCF-DA **(A)** or 5 μM MitoSOX™ Red Mitochondrial Superoxide Indicator **(B)** for 30 min. **(A**, *right*) Quantitative analysis of DCF fluorescence intensity. **(B**, *right*) Quantitative analysis of mitochondrial ROS (MitoSOX) fluorescence intensity. **(C)** Representative photomicrographs show macrophage ingestions of apoptotic cells. Bronchoalveolar lavage was performed, cytospins were stained, and alveolar macrophage ingestions of apoptotic cells were quantified by calculating a phagocytic index. **(D)** Green color represents apoptotic cells engulfed by alveolar macrophages. Alveolar macrophages were cultured ex vivo with apoptotic Jurkat cells labeled with PKH67 (green) for 90 min, and phagocytosis was quantified by calculating a phagocytic index. **(C, D)** Original magnification: 200 ×. Scale bars = 20 μm. Arrows indicate alveolar macrophages with engulfed apoptotic cells or fragments. **(E, F)** ELISA was performed to quantify the abundance of TGF-β1 in culture supernatants of alveolar macrophages **(E)** and bronchoalveolar lavage fluid (BALF) **(F)**. WT and *AIM* ^−/−^ mice were intraperitoneally administered rGas6 (50 μg/kg) 1 day before intratracheal instillation with LPS (4.5 mg/kg) and then once daily thereafter. Animals were euthanized on day 3 after LPS challenge. Values represent the mean ± standard error of three **(A-D)** or five **(E, F)** mice per group. NS, not significant; *P < 0.05, **P < 0.01, and ***P < 0.001 compared with saline control or as indicated.

Elevated ROS levels and autophagy are strongly associated with pulmonary diseases (50). Previously, we demonstrated that AIM reduced mtROS generation in BMDMs stimulated with LPS/ATP (46). Here, we examined ROS generation in alveolar macrophages from WT mice at day 3 post-LPS treatment using H2DCFDA fluorescence and MitoSOX red mitochondrial superoxide indicator. We found that rGas6 suppressed the enhanced ROS and mtROS generation induced by LPS treatment in alveolar macrophages from WT mice but not in those from *AIM^−/−^* mice (**Figures 6A, B**).

We previously reported that oxidant stress negatively modulates efferocytosis during intense lung inflammation (34). Therefore, we examined the role of AIM production in the efferocytic activity of alveolar macrophages at day 3 post-LPS treatment. Similar to previous findings (34, 51), phagocytic indices in alveolar macrophages from mice treated with LPS alone were enhanced compared with those from control mice treated with saline (**Figure 6C**). Administration of rGas6 enhanced the efferocytic activity of alveolar macrophages at day 3 post-LPS treatment. In addition, we assessed ex vivo the phagocytic activities of alveolar macrophages obtained at day 3 post-LPS treatment. Freshly obtained alveolar macrophages from mice treated with saline, LPS, or LPS and rGas6 were co-cultured with apoptotic human Jurkat T cells labeled with PKH67 (green). We found that alveolar macrophages taken from mice at day 3 post-LPS treatment had an enhanced phagocytic index compared with those taken from control mice (**Figure 6D**). Administration of rGas6 further enhanced the ability of the alveolar macrophages to phagocytose apoptotic cells ex vivo; however, this effect was not shown in macrophages from AIM-deficient mice.

*In vitro* and *in vivo* experiments showed that ingestion of apoptotic cells by macrophages in LPS-stimulated lungs suppresses proinflammatory processes while upregulating the production of TGF-β1, an anti-inflammatory cytokine (39, 52, 53). Therefore, we examined the role of the Gas6–AIM axis in TGF-β1 production at day 3 post-LPS treatment. The levels of TGF-β1 in BALF and culture supernatants of alveolar macrophages from WT mice treated with LPS and rGas6 were increased compared with those from WT mice treated with LPS alone (**Figures 6E, F**); however, these effects were not observed in AIM-deficient mice.

## Discussion

This study provides important insights into the anti-inflammatory mechanisms of Gas6 signaling and its dependence on AIM production for resolving inflammation in ALI. Our findings suggest that Gas6-induced AIM production orchestrates a multifaceted response involving NLRP3 inflammasome inhibition, autophagy enhancement, ROS suppression, and efferocytosis promotion. These findings not only confirm the protective role of Gas6 in inflammatory conditions but also expand our understanding of the Gas6–AIM axis as a therapeutic target in ALI.

Our results suggest that AIM is essential for the anti-inflammatory effects of Gas6 in ALI. While Gas6 is well-documented for its interaction with TAM receptors to modulate inflammatory signaling (19, 20, 25), this study identifies AIM as a critical downstream effector of Gas6’s actions. The absence of Gas6-induced inflammation resolution in AIM-deficient mice underscores the pivotal role of AIM in this context. These findings align with and extend previous studies reporting the role of AIM in suppressing macrophage-mediated inflammatory responses and promoting tissue repair in various pathological conditions (29–32).

Our findings highlight the dynamic role of Gas6 in modulating inflammation during ALI via downregulating NLRP3 inflammasome activation. Our results emphasize the Gas6–AIM axis’s unique role in inflammasome regulation. Notably, rGas6 reduced IL-1β and IL-18 production and ASC speck formation on day 3 following LPS treatment in WT mice but not in AIM-deficient mice. Furthermore, Gas6 did not alter the transcription of IL-1β or Nlrp3, suggesting its effects are mediated via post-transcriptional mechanisms involving AIM-dependent suppression of inflammasome activation. These findings refine our understanding of Gas6’s anti-inflammatory actions, positioning the Gas6–AIM axis as a critical regulator in the transition from inflammation to resolution. Future research should explore its interaction with other pathways and its therapeutic potential for targeting inflammasomes in ALI and other inflammatory conditions.

Our *in vitro* experiments highlight the critical role of AIM in mediating the effects of Gas6 signaling. Silencing Axl, LXRα, or LXRβ in BMDMs reversed rGas6-induced suppression of IL-1β, IL-18, and caspase-1 activity following LPS/ATP stimulation. This finding indicates that Gas6–Axl–LXRα/β signaling is essential for suppressing NLRP3 inflammasome activation in macrophages. Notably, rAIM treatment retained these inhibitory effects, even in the absence of Gas6–Axl–LXRα/β signaling. These findings confirm that Gas6–Axl–LXRα/β signaling inhibits NLRP3 inflammasome activation by inducing AIM production in macrophages, establishing AIM as an indispensable downstream effector in this pathway.

In LPS-induced inflammation, autophagy and inflammasome activation are closely interconnected processes. Autophagy acts as a negative regulator, mitigating excessive inflammasome activity and preventing hyperinflammation (13). Conversely, impaired autophagy can amplify inflammasome-driven inflammation, underscoring the importance of maintaining balance between these pathways for effective resolution. Our study suggests that rGas6 administration enhances autophagy in alveolar macrophages and lung tissues via an AIM-dependent mechanism, which could contribute to the modulation of NLRP3 activation and support the resolution of inflammation. However, we also acknowledge that LPS alone strongly induces NLRP3 activation and autophagy independently of Gas6, as shown in our findings. Likewise, our data suggest that certain aspects of NLRP3 inflammasome activation and autophagy operate independently of both Gas6 and AIM. These results emphasize the complex interplay between autophagy and inflammasome activation, which involves both overlapping and distinct regulatory mechanisms. Further research is required to clarify the independent regulatory mechanisms of these processes and their interaction with Gas6 and AIM, which may provide valuable insights for the development of targeted therapeutic strategies to treat inflammatory diseases.

ROS, particularly mtROS, are recognized as key activators of the NLRP3 inflammasome (54, 55). Previous studies have shown that AIM suppresses ROS generation in BMDMs stimulated with LPS and ATP (35). Our findings extend these observations, demonstrating that rGas6 administration suppresses ROS production in an AIM-dependent manner in the context of *in vivo* LPS treatment. This Gas6-induced AIM-mediated suppression of ROS may play role in modulating the inhibition of NLRP3 inflammasome activation and reducing oxidative stress, which could help mitigate tissue damage associated with ALI.

Efferocytosis, the clearance of apoptotic cells, is a vital process for resolving inflammation and promoting macrophage polarization to an anti-inflammatory M2 phenotype (56). Gas6 has long been known to enhance efferocytosis through its interaction with TAM receptors (57–59). Our study demonstrates that AIM is indispensable for these effects, with rGas6 failing to enhance efferocytosis in AIM-deficient mice. AIM facilitates efferocytosis by upregulating the expression of Mer, a key receptor involved in the clearance of apoptotic cells. This regulation occurs through an autophagy-mediated pathway, where AIM induces ID3, a transcription factor essential for Mer expression. Silencing ATG7, a key autophagy gene, or ID3 inhibits Mer expression and subsequently reduces efferocytic activity. These findings underscore the role of AIM in facilitating efferocytosis by modulating Mer expression through autophagy-dependent mechanisms (32, 58). Furthermore, recent studies have highlighted the interconnected relationship between autophagy and efferocytosis (60–63). AIM appears to play a central role in coordinating these processes, linking phagocytic activity with cellular mechanisms that regulate inflammation resolution and tissue repair. Further studies are needed to delineate the precise molecular interactions between AIM and TAM receptors and to explore AIM’s role in integrating autophagy and efferocytosis pathways.

The therapeutic implications of these findings are significant. AIM’s role as a mediator of Gas6’s anti-inflammatory effects positions it as a promising target for interventions in ALI and other inflammatory diseases. Enhancing AIM production could provide a multifaceted therapeutic approach, addressing key inflammatory pathways such as inflammasome activation, ROS generation, autophagy regulation, and efferocytosis.

In addition to the Gas6–AIM–NLRP3 axis, several other molecular pathways may contribute to macrophage function and inflammasome regulation. Syk and PLCγ signaling, downstream of TAM receptors, are involved in immune cell activation and may influence autophagy and inflammasome modulation (64, 65). Glycogen synthase kinase-3 beta (GSK3β), through its regulation of β-catenin, suppresses pro-inflammatory signaling, potentially complementing the anti-inflammatory effects of TAM receptor activation (66, 67). The link between Gas6 and LXRα/β activation suggests an intersection with lipid metabolism, which could influence macrophage polarization and efferocytosis (68). Additionally, Gas6’s interaction with phosphatidylserine on apoptotic cells may involve other receptors like T-cell immunoglobulin and mucin domain containing 4 (TIM4) and brain-specific angiogenesis inhibitor 1 (BAI1), enhancing apoptotic cell clearance (69). These pathways highlight the complexity of macrophage signaling and suggest additional mechanisms through which Gas6 may regulate inflammation and tissue repair.

Future studies should explore the translational potential of AIM-targeted therapies, such as AIM mimetics or strategies to upregulate endogenous AIM production. Additionally, investigating whether these mechanisms operate in other inflammatory or autoimmune diseases could broaden the therapeutic scope of the Gas6-AIM axis.

## Conclusion

In conclusion, this study highlights the critical role of Gas6-induced AIM production in resolving inflammation in LPS-induced ALI. AIM facilitates inflammation resolution through multiple interconnected mechanisms, including NLRP3 inflammasome inhibition, ROS suppression, autophagy promotion, and efferocytosis enhancement. These findings provide a strong foundation for targeting the Gas6–AIM axis as a novel therapeutic strategy for ALI and related inflammatory diseases.

## Data Availability

The original contributions presented in the study are included in the article/**Supplementary Material**, further inquiries can be directed to the corresponding author/s.
