# Supplementary material for: Gas6 induces AIM to suppress acute lung injury in mice by inhibiting NLRP3 inflammasome activation and inducing autophagy
Source: Front Immunol. 2025 Feb 17;16:1523166. doi: 10.3389/fimmu.2025.1523166 (PMC11873840; doi:10.3389/fimmu.2025.1523166)

Figure 2E

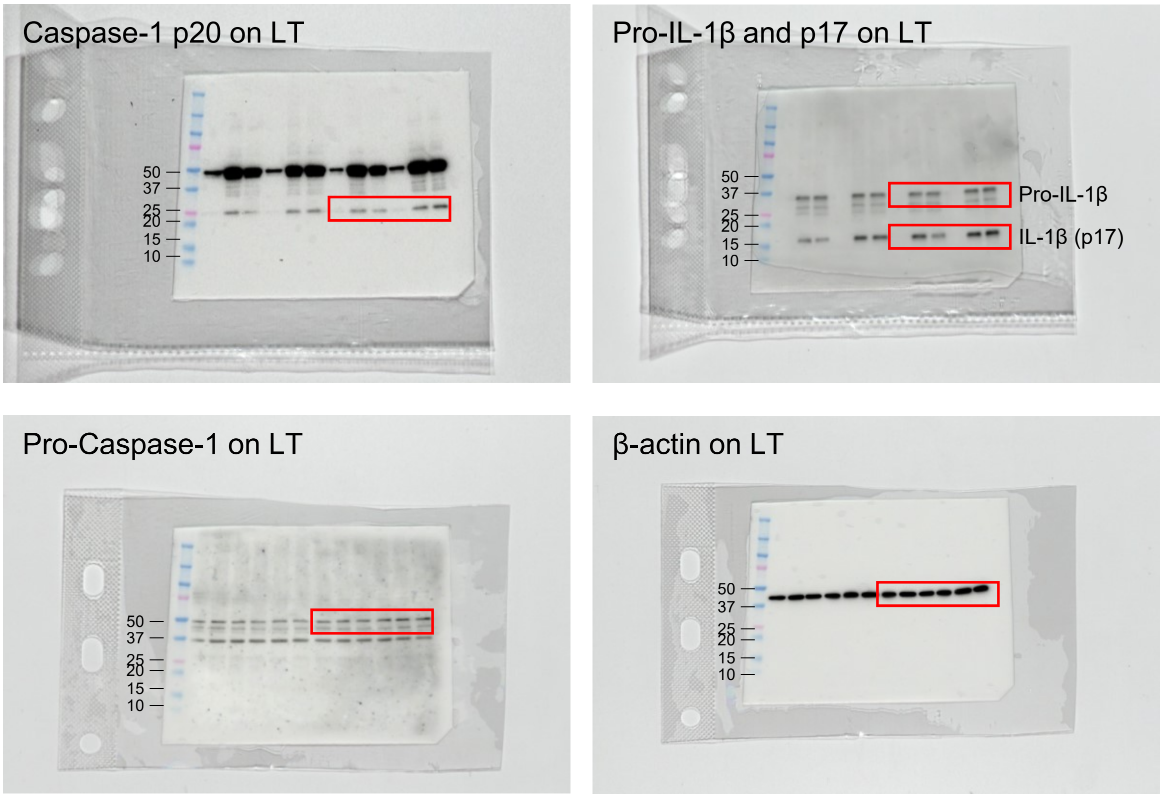

Figure 4B

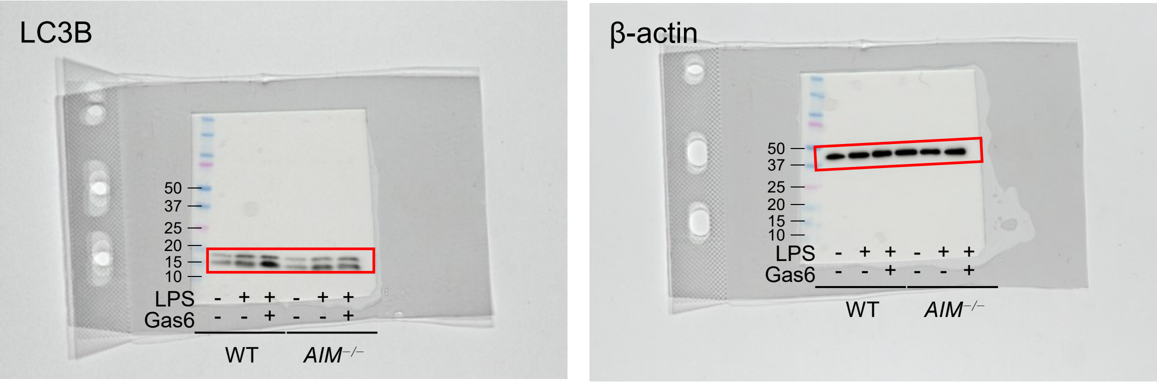

Figure 5A

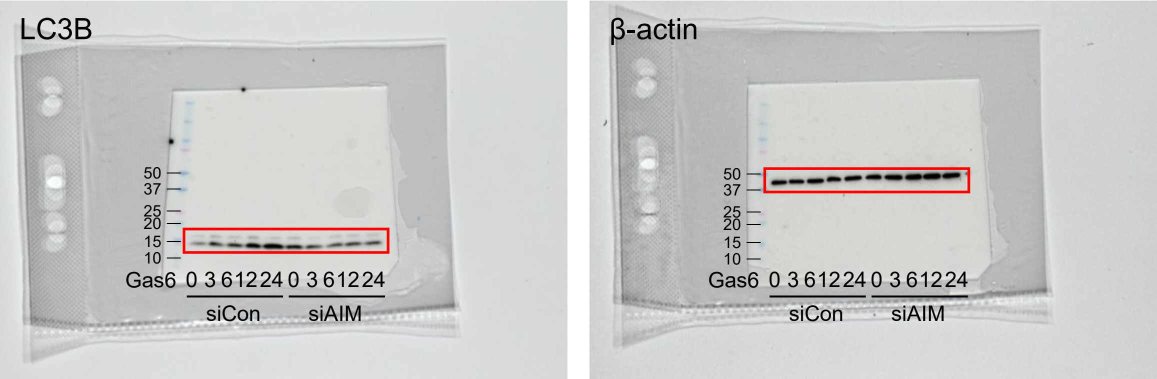

Figure S1A

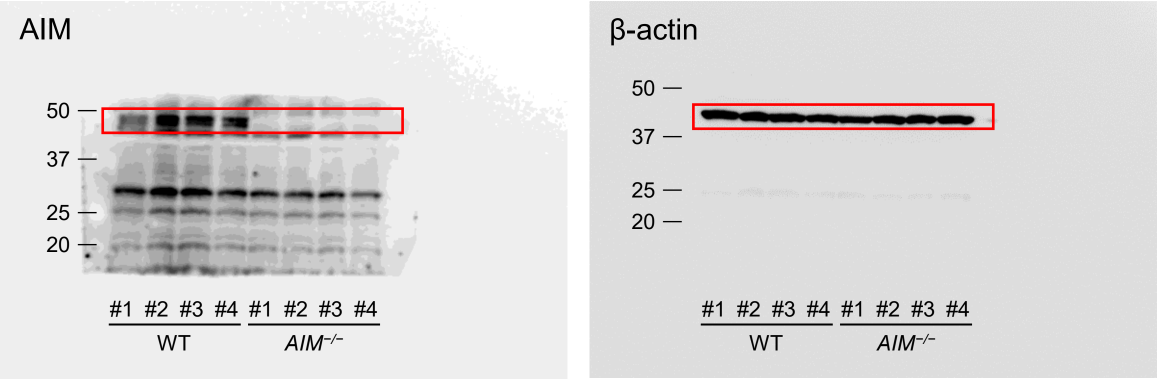

Figure S2D

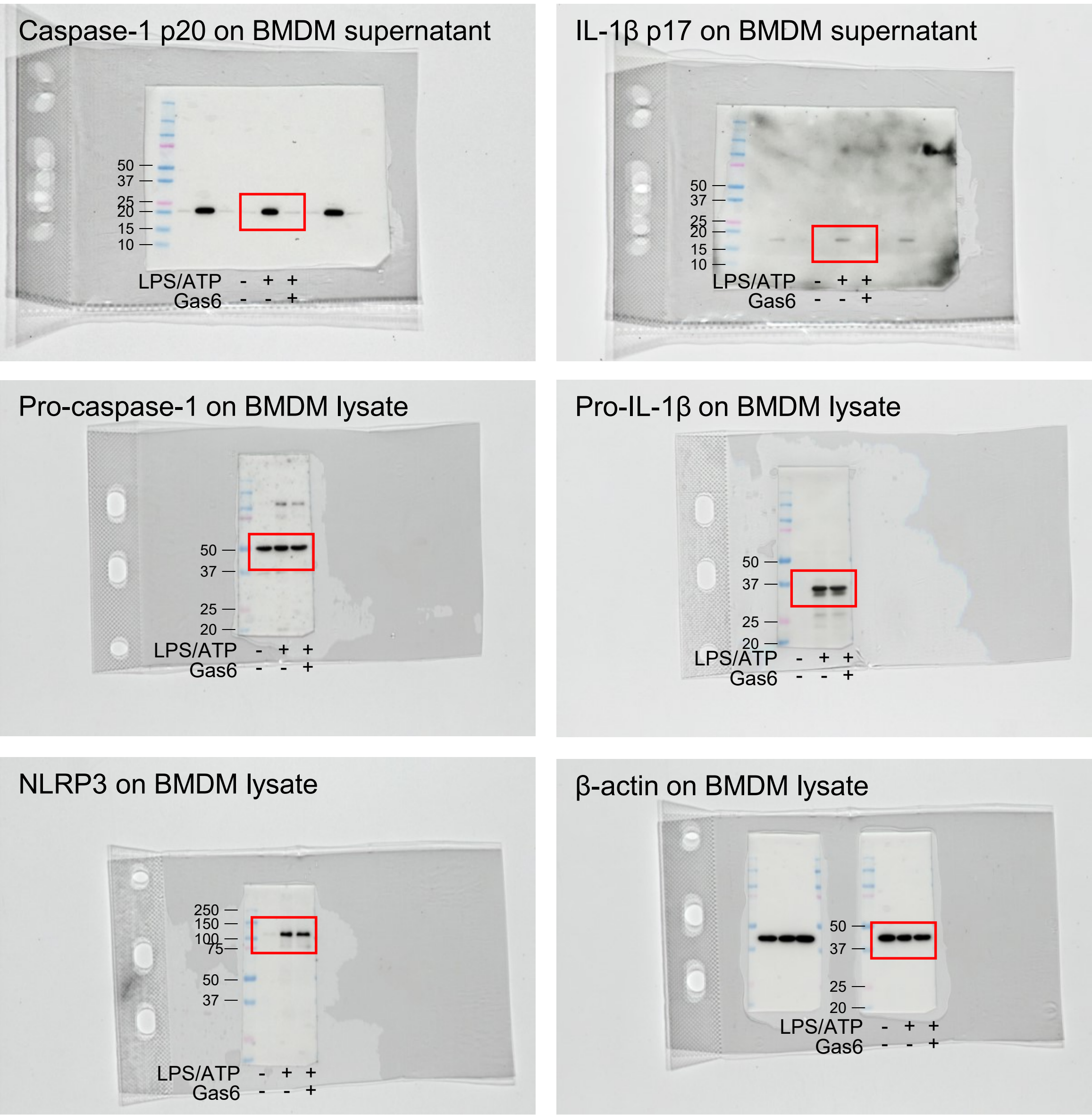

Figure S3A

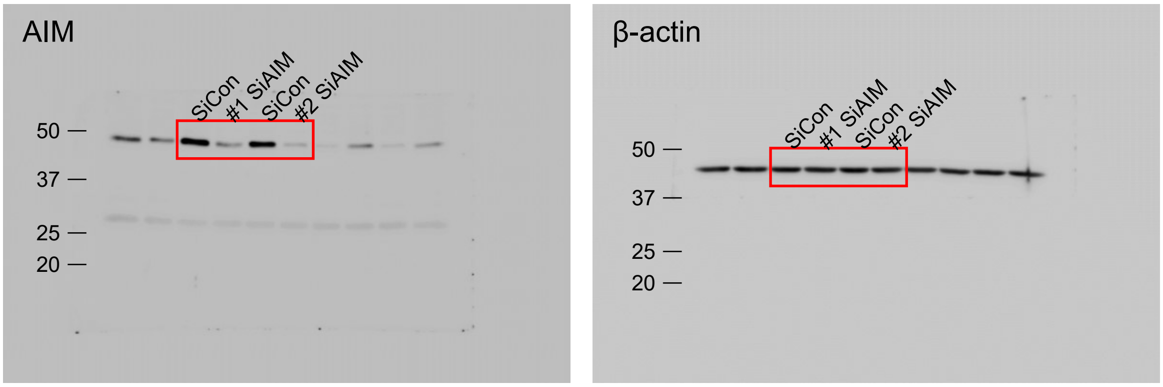

Figure S3B

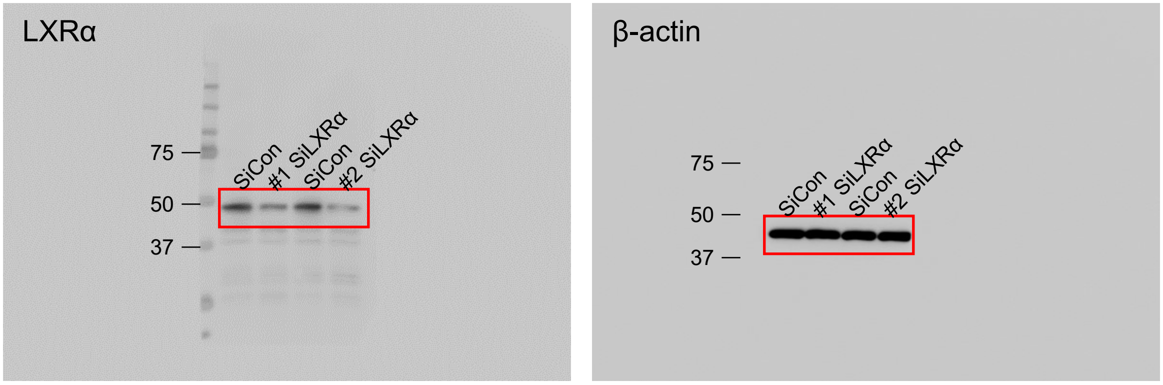

Figure S3C

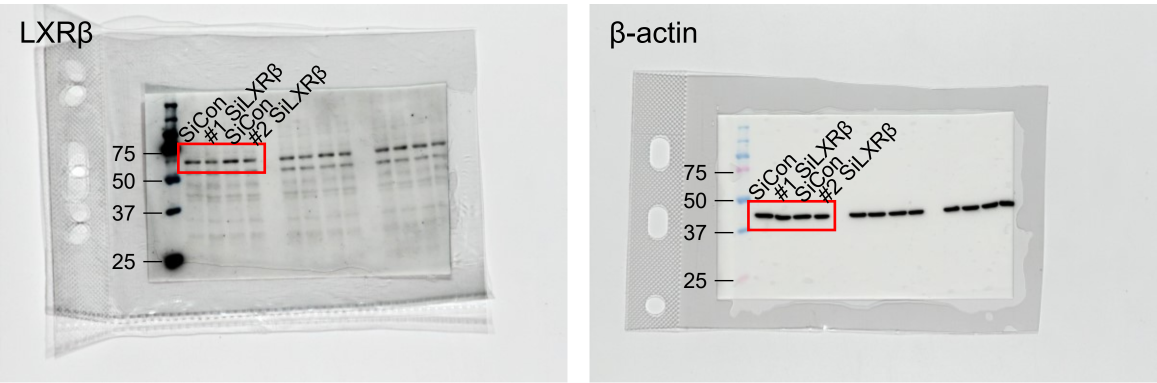

Figure S3D

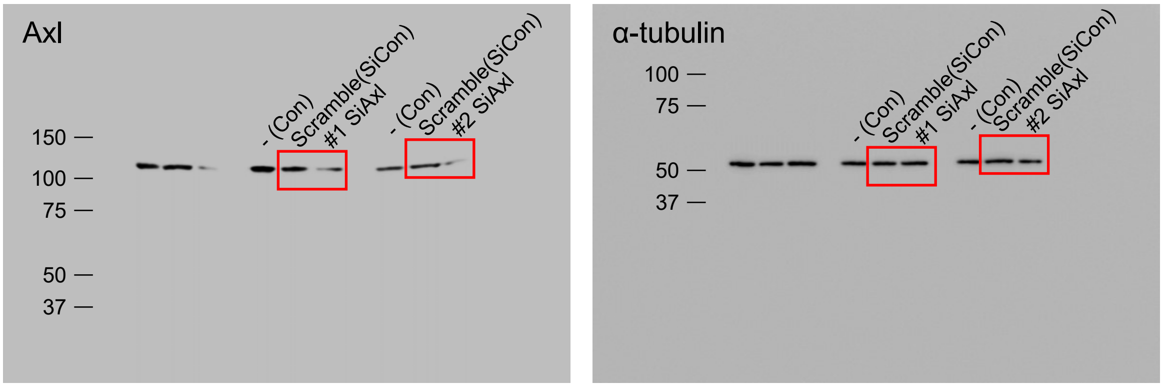

Figure S4D

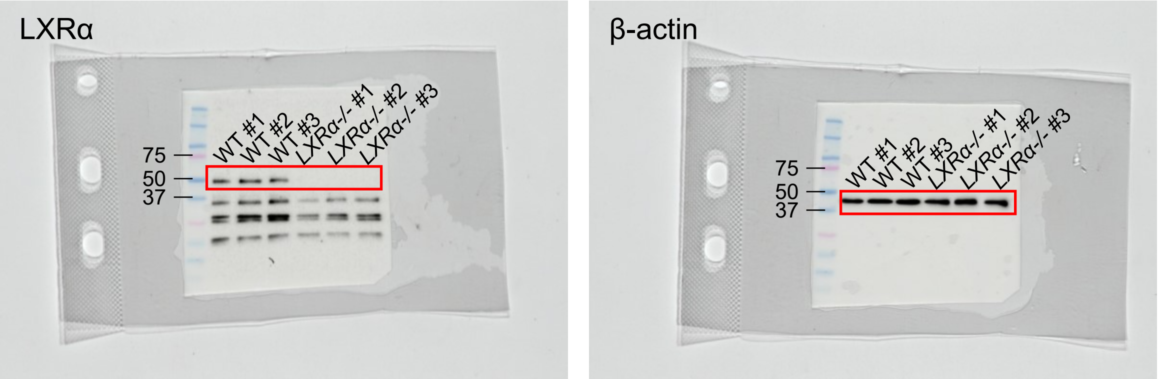

Figure S5B

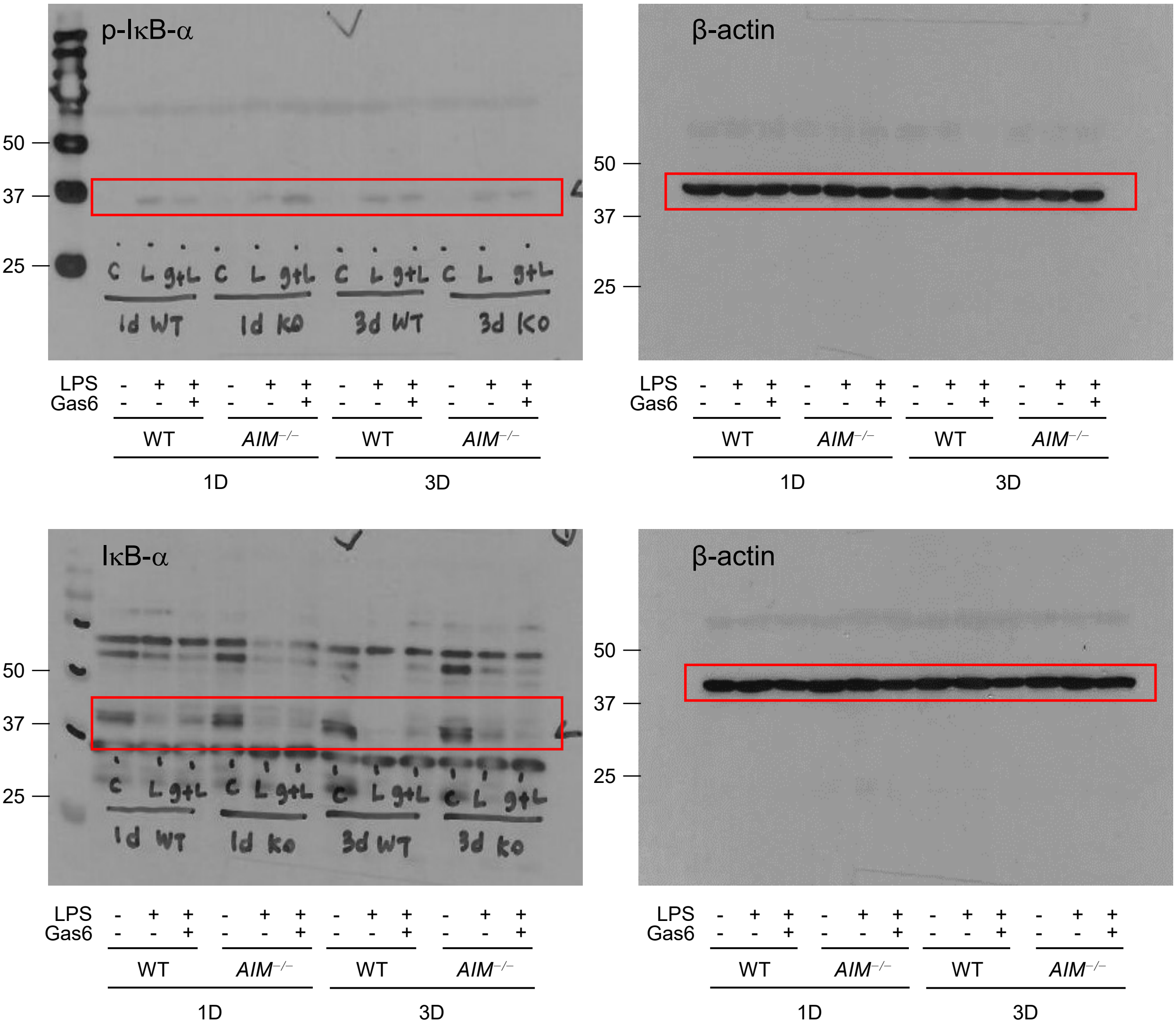

Supplement: Supplementary file 1 [file DataSheet1.pdf]
