# Supplementary material for: Gas6 induces AIM to suppress acute lung injury in mice by inhibiting NLRP3 inflammasome activation and inducing autophagy
Source: Front Immunol. 2025 Feb 17;16:1523166. doi: 10.3389/fimmu.2025.1523166 (PMC11873840; doi:10.3389/fimmu.2025.1523166)
Supplement: Supplementary file 2 [file DataSheet2.pdf]

Supplementary information

Gas6 induces AIM to suppress acute lung injury in mice by inhibiting NLRP3 inflammasome activation and inducing autophagy

Seonghee Jeong, Kyungwon Yang, Ye-Ji Lee, Joo-Won Park, Eun-Mi Park, and Jihee Lee Kang

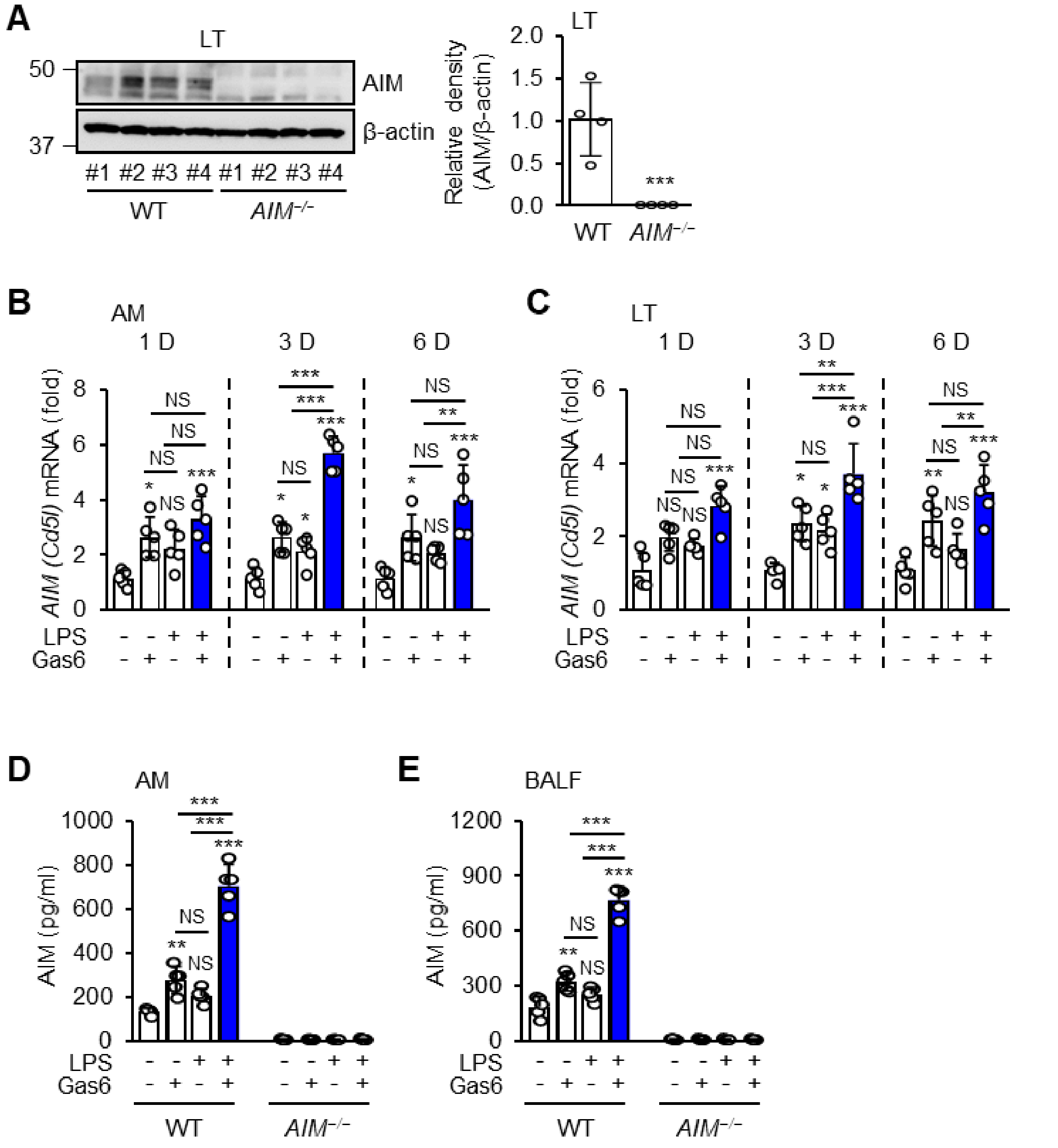

Supplementary Figure 1. rGas6 enhances AIM production in lungs after LPS treatment. (**A**) Left: Immunoblot analysis of AIM in lung tissue from wild type (WT) and *AIM*$^{-/-}$ mice (n = 4 mice). Right: The relative densitometric intensity was determined for each band and normalized to β-actin. (**B**, **C**) qRT-PCR analysis of *AIM* (*Cd5l*) mRNA levels in alveolar macrophages and lung tissue from WT mice on days 1, 3, or 6 after LPS treatment (n = 5 mice). (**D**, **E**) ELISA was performed to quantify AIM in culture supernatants of alveolar macrophages and bronchoalveolar

lavage fluid (BALF) from WT and *AIM*$^{-/-}$ mice at 3 days after LPS treatment (n = 5 mice). (**B–E**) Where indicated, WT and *AIM*$^{-/-}$ mice were intraperitoneally administered rGas6 (50 μg/kg) 1 day before intratracheal instillation with LPS (4.5 mg/kg), and then once daily thereafter. Values represent the mean ± standard error of four (**A**) or five mice (**B–E**) per group. ns: not significant; *$P < 0.05$, **$P < 0.01$, and ***$P < 0.001$ compared with saline control or as indicated.

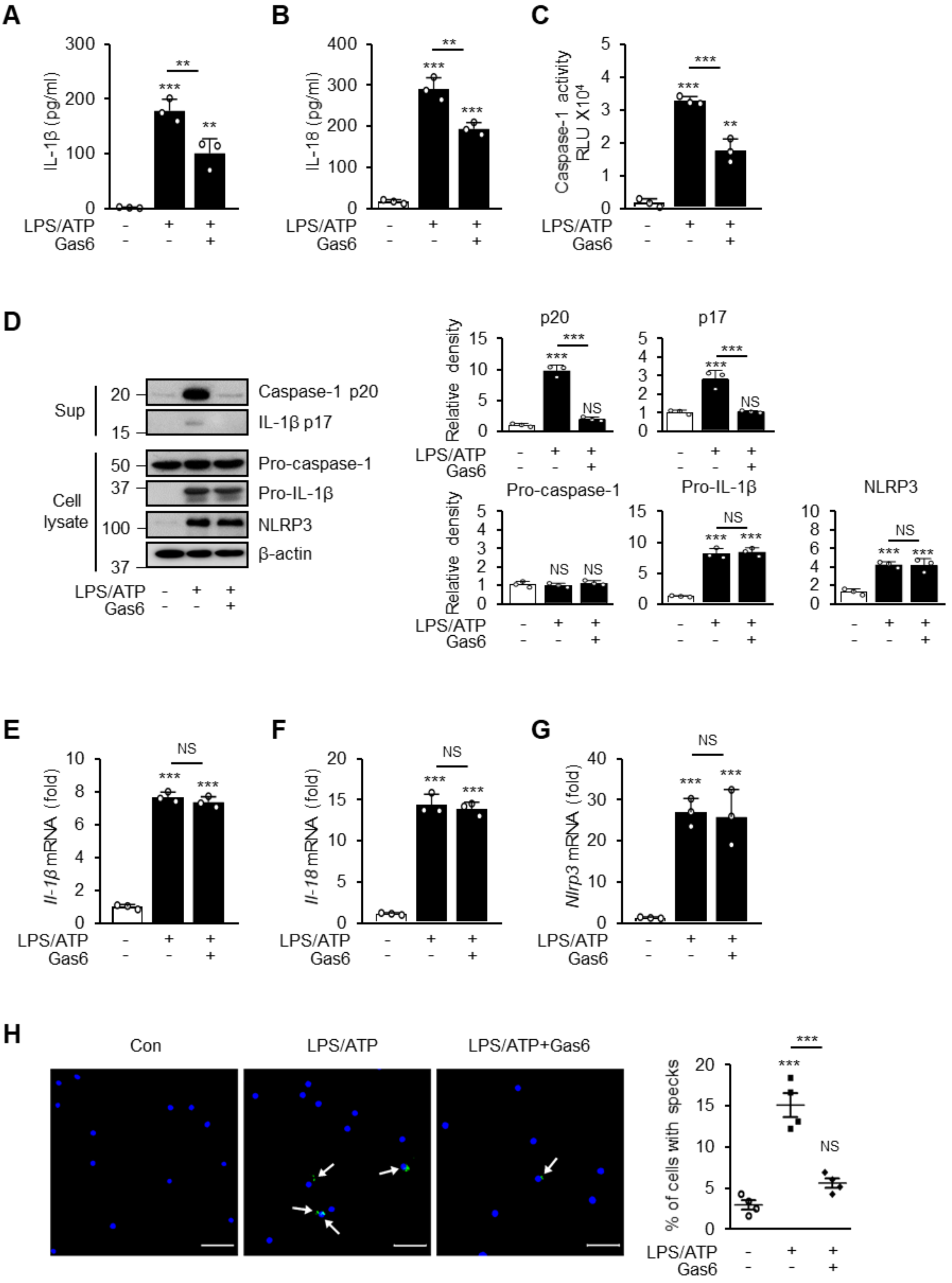

Supplementary Figure 2. rGas6 inhibits NLRP-3 inflammasome activation in BMDMs. Levels of secreted IL-1β (**A**), IL-18 (**B**) in culture supernatants of mouse bone marrow-derived macrophages (BMDMs) were measured using ELISA. (**C**)

Caspase-1 activity was measured in culture supernatants of BMDMs. (**D**) Left: Immunoblot analysis of the indicated protein in supernatants and lysates of BMDMs. Right: The relative densitometric intensity was determined for each band and normalized to β-actin. (**E–G**) qRT-PCR analysis of *IL-1β*, *IL-18*, and *Nlrp3* mRNA levels in BMDMs. (**H**) Left: Representative immunofluorescence confocal microscopic images of ASC specks. ASC in green. Arrows point to ASC specks. Original magnification: 400 ×. Scale bars: 50 μm. Right: Quantification of the percentage of cells with ASC specks (4 × 200 cells/nuclei [DAPI-stained], analyzed with ImageJ). (**A–H**) BMDMs were treated with rGas6 (100 ng/ml) treatment for 24 h, followed by LPS (100 ng/ml) for 4 h and ATP (1 mM in **A–G** or 5 mM in **h**) for 1 h. Values represent the mean ± standard error of three (**A–G**) or four (**H**) independent experiments. ns: not significant; $^{**}P < 0.01$, and $^{***}P < 0.001$ compared with control or as indicated.

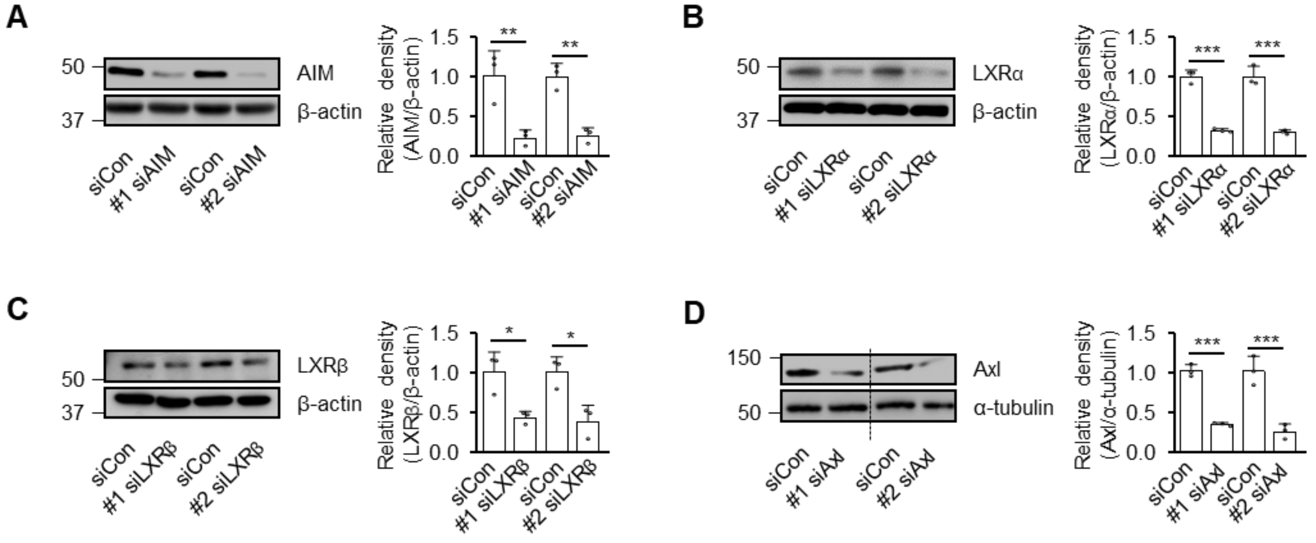

Supplementary Figure 3. Knockdown of AIM, LXRα, LXRβ, and Axl was achieved by transfecting BMDMs with specific siRNAs. Left: Immunoblot analysis of AIM (**A**), LXRα (**B**), LXRβ (**C**), and Axl (**D**) in BMDMs transfected with two types of siRNAs for each target. Right: Densitometric analysis of the relative abundance of AIM, LXRα, LXRβ, and Axl (lower). Values represent the mean ± standard error of three independent experiments. **P* < 0.05, ***P* < 0.01, and ****P* < 0.001 as indicated.

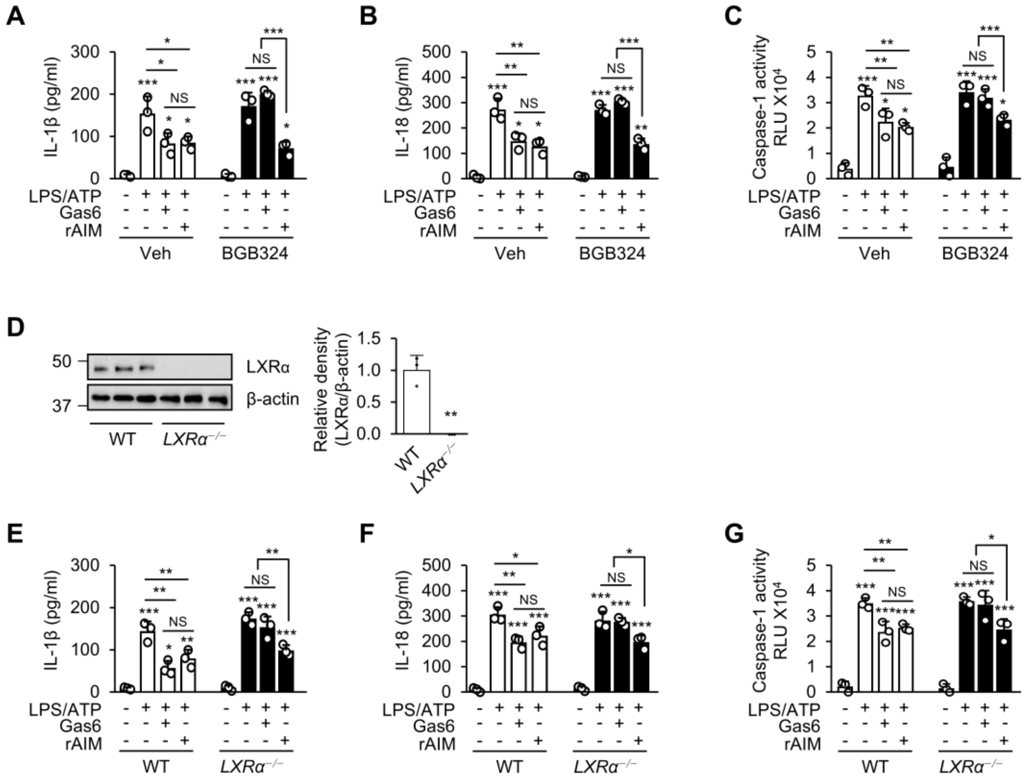

Supplementary Figure 4. Gas6-mediated inhibition of NLRP3 inflammasome activation is reversed by Axl signaling inhibition and in LXRα-deficient BMDMs. ELISA was performed to quantify the abundance of IL-1β (**A**, **E**) and IL-18 (**B**, **F**) in the culture supernatants of bone marrow-derived macrophages (BMDMs). (**C**, **G**) Caspase-1 activity was assessed in culture supernatants. (**D**) Left: Immunoblot analysis of LXRα in BMDMs from wild type (WT) and *LXRα*$^{-/-}$ mice (n = 3 mice). Right: The relative densitometric intensity was determined for each band and normalized to β-actin. (**A-C**) BMDMs were pretreated with the Axl-specific inhibitor BGB 324 (1 μg/ml) or (**E-G**) BMDMs from *LXRα*$^{-/-}$ and WT mice were treated with rGas6 (100 ng/ml) or rAIM (1 μg/ml) for 24 h, followed by stimulation with LPS (100 ng/ml) for 4 h and ATP (1 mM) for 1 h. Values represent the mean ± standard error of three independent experiments. ns: not significant; *P < 0.05, **P < 0.01, and ***P < 0.001 compared with control or as indicated.

Supplementary Table 1. List of antibodies used for this study

Antigen	Vendor	Cat. No.	Source	Species cross-reactivity	Application	Dilution
AIM	R&D system	AF2834	Goat	M	WB	1:2000
ASC	Adipogen Life Science	AF0006	Rabbit	H M	ICC	1:2000
ATG5	Invitrogen	PA1-46178	Rabbit	H M R B	IHC	1:100
α-tubulin	Sigma	T6199	Mouse	H M R	WB	1:5000
β-actin	Santa-Cruz Biotechnology	Sc-69879	Mouse	M	WB	1:5000
Caspase-1 (p20)	Adipogen Life Science	AG-20B-0042	Mouse	M R	WB	1:2000
F4/80	Invitrogen	14-4801-82	Rat	H M R	IHC	1:50
IL-1β	Cell Signaling Technology	12507	Rabbit	M	WB	1:2000
LC3B	Cell Signaling Technology	83506	Mouse	H M R	WB	1:2000
Pro-caspase-1	Cell Signaling Technology	2225	Rabbit	H	WB	1:1000

* Abbreviation: ICC-Immunocytochemistry, IHC-Immunohistochemistry, WB-Western blot; H-Human, M-Mouse, R-Rat, B-Bovine, Mk-Monkey

Supplementary Table 2. Sequences of qRT-PCR primer

murine gene	forward (5' → 3')	reverse (5' → 3')
36B4	GCTTCGTGTTCACCAAGGAGGA	GTCCTAGACCAGTGTTCTGAGC
Atg5	AAGTCTGTCCTTCCGCAGTC	TGAAGAAAGTTATCTGGGTAGCTCA
Atg7	CCTGTGAGCTTGGATCAAAGGC	GAGCAAGGAGACCAGAACAGTG
Beclin1	AGGATGGTGTCTCTCGAAGATT	GATCAGAGTGAAGCTATTAGCACTTTC
Cd5l (AIM)	GAGGACACATGGATGGAATGT	ACCCTTGTGTAGCACCTCCA
Il18	GACAGCCTGTGTTCGAGGATATG	TGTTCTTACAGGAGAGGGTAGAC
Il1b	AAATACCTGTGGCCTTGGGC	CTTGGGATCCACACTCTCCAG
Map1lc3b	CCCCACCAAGATCCCAGT	CGCTCATGTTCACGTGGT
Nlrp3	TCTAGAGGACCTTGAAGATG	AAGTGATCTGCCTTCTCCAT

Supplementary Table 3. List of siRNA

murine gene	Sense	Antisense
Axl #1	GAGAUGGACAGAUCCUAGA	UCUAGGAUCUGUCCAUCUC
Axl #2	CACACACUCAAGAAUCCAATT	UUGGAUUCUUGAGUGUGUGTT
Cd5l (AIM) #1	AAGAUGUCGUGUUCUGGACAA	UUGUCCAGAACACGACAUCUU
Cd5l (AIM) #2	AACGGAAGACACGUUGGCUCA	UGAGCCAACGUGUCUUCCGUU
Lxrα #1	CGUAGCAUUAAGGGAGAGU	ACUCUCCCUUAAUGCUACG
Lxrα #2	GACAGAGCUUCGUCCACAA	UUGUGGACGAAGCUCUGUC
Lxrβ #1	ACGCUUACACCUCAGCCUA	UAGGCUGAGGUGUGUAAGCGU
Lxrβ #2	GCCACGUCACCCACUAUUA	UAAUAGUGGGUGACGUGGC
